# Chaperone‐Mediated Autophagic Degradation of USP9X in Macrophages Exacerbates Postmyocardial Infarction Inflammation and Cardiac Dysfunction

**DOI:** 10.1002/advs.202518950

**Published:** 2026-01-28

**Authors:** Biqing Wang, Xiangheng Cai, Mengqi Li, Xue Liu, Junhui Xue, Ye Liu, Ding Ai, Xinyang Hu

**Affiliations:** ^1^ Department of Cardiology The Second Affiliated Hospital Zhejiang University School of Medicine Zhejiang University Hangzhou China; ^2^ State Key Laboratory of Transvascular Implantation Devices Heart Regeneration and Repair Key Laboratory of Zhejiang province Transvascular Implant Instrument Research Institute Binjiang Institute of Zhejiang University Hangzhou China; ^3^ The Affiliated Hospital of Qingdao University Qingdao Shandong China; ^4^ Tianjin Medical University General Hospital Tianjin China; ^5^ Zhejiang Chinese Medical University Hangzhou China; ^6^ The Province and Ministry Co‐sponsored Collaborative Innovation Center for Medical Epigenetics State Key Laboratory of Experimental Hematology National Clinical Research Center for Blood Diseases Key Laboratory of Immune Microenvironment and Disease (Ministry of Education) Tianjin Institute of Cardiology The Second Hospital of Tianjin Medical University Tianjin Medical University Tianjin China

**Keywords:** chaperone‐mediated autophagy, inflammation, macrophage polarization, proteostasis, protein translational modifications, ventricular remodeling

## Abstract

Excessive macrophage‐mediated inflammation following myocardial infarction (MI) exacerbates infarct expansion and impairs cardiac repair; however, the regulatory mechanisms remain poorly understood. Here, it is reported that ubiquitin‐specific peptidase 9 X‐linked (USP9X) was significantly downregulated in macrophages during early post‐MI inflammation. Macrophage‐specific deficiency of USP9X enhanced expression of pro‐inflammatory genes, thereby impeding cardiac functional recovery. Mechanistically, USP9X deubiquitinated and stabilized tumor necrosis factor receptor‐associated factor (TRAF)‐type zinc finger domain containing 1 (TRAFD1), a negative regulator of Toll‐like receptor (TLR) signaling, thereby restraining inflammatory responses. Moreover, inflammatory stimuli triggered acetylation of USP9X at K2414, exposing a latent KFERQ motif that promoted its recognition by the molecular chaperone heat shock cognate protein 70 (HSC70) and facilitated subsequent lysosomal degradation via chaperone‐mediated autophagy (CMA). Consistently, both genetic inhibition of HSC70 and pharmacological blockade of lysosomal degradation prevented USP9X degradation following inflammatory stimulation. Furthermore, a cell‐penetrating peptide mimicking the KFERQ sequence of USP9X that blocked its interaction with HSC70 and the subsequent CMA‐mediated degradation, thereby promoting inflammation resolution and cardiac repair post‐MI. Collectively, these findings establish the USP9X–TRAFD1 axis and its CMA‐mediated degradation as critical checkpoints in post‐MI inflammation, highlighting USP9X stabilization as a therapeutic strategy for ischemic heart disease.

## Graphical abstract

This study demonstrates that inflammatory stimuli induce the acetylation‐triggered, chaperone‐mediated autophagic degradation of ubiquitin‐specific peptidase 9 X‐linked (USP9X) in macrophages. USP9X acts as a macrophage “inflammation switch” after myocardial infarction (MI). USP9X loss destabilizes tumor necrosis factor receptor‐associated factor (TRAF)‐type zinc finger domain containing 1 (TRAFD1), exacerbating inflammation and impairing cardiac repair post‐MI. A cell‐penetrating peptide disrupting the USP9X–HSC70 interaction preserves USP9X expression, restrains inflammation, and highlights therapeutic potential for ischemic heart disease.

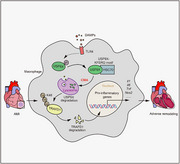

## 1 Introduction

Ischemic heart disease, particularly acute myocardial infarction (AMI), remains one of the leading causes of death worldwide [1, 2, 3]. While reperfusion therapies have significantly reduced early mortality rates, the incidence of post‐MI heart failure continues to rise, underscoring the unmet need for therapies that reduce adverse ventricular remodeling [4, 5].

Following AMI, the cardiac inflammatory response initiates ventricular remodeling [6, 7]. Macrophages are key mediators of the early inflammatory response, whose infiltration and activation in the infarcted region are critical for clearing necrotic tissue [8]. Their transition from a pro‐inflammatory to an anti‐inflammatory phenotype is crucial for orchestrating tissue repair [9]. However, uncontrolled and prolonged inflammation can exacerbate myocardial injury and promote adverse ventricular remodeling [10]. Therefore, modulating macrophage polarization represents a critical therapeutic strategy for managing postinfarction inflammation.

Emerging evidence suggests the indispensable role of deubiquitinating enzymes (DUBs) in AMI pathogenesis and subsequent cardiac remodeling. Elevated ubiquitin immunoreactivity in lesions from patients with acute coronary syndromes suggests altered DUB activity during AMI onset [11]. Furthermore, several DUBs have been implicated in post‐MI cardiac remodeling, affecting cardiomyocyte survival and promoting fibrosis [12, 13, 14, 15, 16]. Although DUBs are known regulators of key immune signaling molecules such as RIG‐I and tumor necrosis factor receptor‐associated factor (TRAF) family proteins [17, 18], their specific roles in regulating the cardiac immune response, particularly within macrophages during AMI, remain poorly defined.

Ubiquitin‐specific peptidase 9 X‐linked (USP9X), an evolutionarily conserved DUB, regulates diverse biological processes including angiogenesis, apoptosis, and immune responses [19, 20, 21, 22]. Our prior work revealed that USP9X downregulation in macrophages promotes atherosclerosis through class A1 scavenger receptor (SR‐A1) ubiquitination [23]. However, the role and regulatory mechanisms of USP9X specifically in macrophage‐driven inflammation during AMI remain largely unexplored.

The dynamic regulation of USP9X post‐MI suggests active regulatory mechanisms controlling its stability. Exploiting cellular protein quality control pathways to modulate disease‐relevant proteins is emerging as a promising therapeutic paradigm [24, 25]. Chaperone‐mediated autophagy (CMA) is a selective lysosomal degradation pathway that maintains proteostasis by targeting specific cytosolic proteins containing a KFERQ‐like pentapeptide motif. In this process, the molecular chaperone heat shock cognate protein 70 (HSC70) recognizes the motif and facilitates the substrate translocation into the lysosome for degradation [26, 27]. Accumulating evidence suggests that CMA plays a significant role in several cardiovascular diseases such as atherosclerosis [28, 29]. In addition, strategies targeting CMA are being explored to restore proteostasis [30, 31]. We therefore hypothesized that CMA might contribute to the degradation of USP9X in macrophages during post‐MI inflammation.

In this study, we aimed to clarify the role of USP9X in macrophage‐mediated inflammation and cardiac repair following AMI. Furthermore, we sought to identify key substrates of USP9X, particularly within immune signaling pathways, that mediate these effects. Finally, we investigated the potential role of CMA in the post‐translational regulation of USP9X. Collectively, our findings aim to define a novel regulatory axis in post‐AMI inflammation and explore the therapeutic potential of modulating the CMA–USP9X pathway to improve cardiac outcomes.

## 2 Results

### 2.1 USP9X Is Identified as a Macrophage Inflammatory Regulator During the Early Phase of MI

**FIGURE 1:**
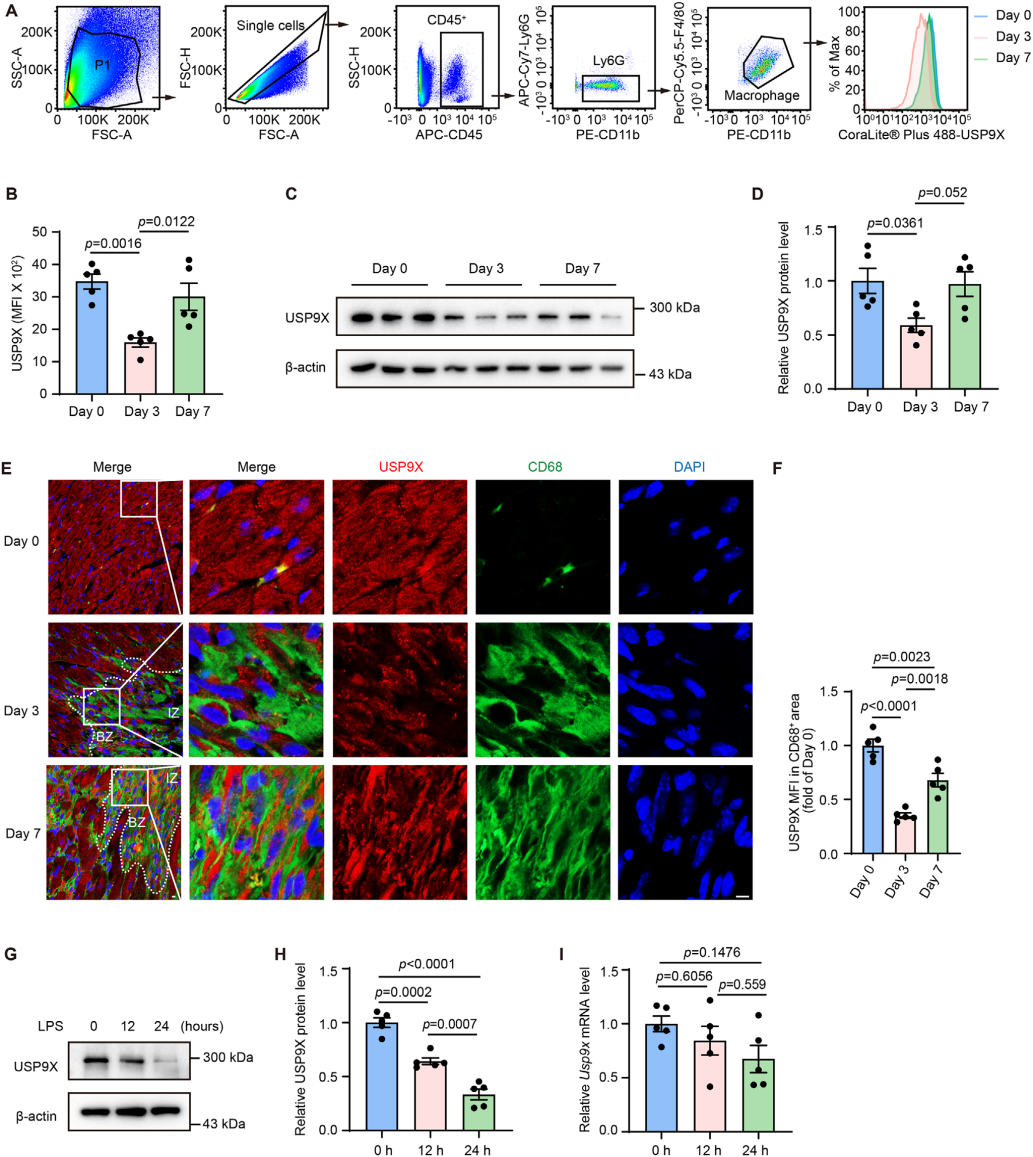
Dynamic regulation of USP9X in macrophages during early MI. (A) Flow‐cytometric analysis of USP9X expression in cardiac macrophages from WT mice at the indicated times after MI. (B) Quantification of USP9X MFI shown in Panel A. Statistical significance was assessed using one‐way analysis of variance (ANOVA) followed by Tukey's multiple comparisons test (*n* = 5). (C) WB analysis of USP9X expression in cardiac macrophages from WT mice at the indicated times after MI. (D) Quantification of USP9X protein levels shown in Panel C. One‐way ANOVA with Tukey's multiple comparisons test (*n* = 5). (E) Representative immunofluorescence staining of USP9X (red), CD68 (green), and 4′,6‐diamidino‐2‐phenylindole (DAPI) (blue) in heart cryosections from WT mice at the indicated time points after MI; IZ: infarct zone; BZ: border zone. Scale bar, 5 µm. (F) Quantification of USP9X MFI within CD68^+^ regions. One‐way ANOVA with Tukey's multiple comparisons test (*n* = 5). (G) WB analysis of USP9X in bone marrow–derived macrophages (BMDMs) at the indicated times after LPS stimulation. (H) Quantification of USP9X levels shown in Panel G. One‐way ANOVA with Tukey's multiple comparisons test (*n* = 5). (I) Quantitative polymerase chain reaction (PCR) was performed to detect the mRNA levels of *Usp9x* in BMDMs at indicated time following LPS stimulation. Target gene expression was normalized to the level of *Actb* mRNA. One‐way ANOVA with Tukey's multiple comparisons test (*n* = 5).

To investigate the dynamic changes in USP9X in macrophages following MI, we induced MI in mice by performing permanent ligation of the left anterior descending artery, thereby mimicking human MI pathology. We assessed the expression of endogenous USP9X in cardiac macrophages from wild‐type (WT) mice at 0, 3, and 7 days post‐MI. Flow‐cytometric analysis showed that USP9X mean fluorescence intensity (MFI) decreased by nearly 50% within the initial 3 days following MI. Subsequently, USP9X expression rebounded moderately by day 7, indicating its involvement in the temporal dynamics of cardiac inflammatory responses during the early phase of MI (Figure 1A,B). Consistently, the western blot (WB) assay revealed that the expression of USP9X in cardiac macrophages exhibited a trend consistent with that observed in flow cytometry, characterized by an initial decrease followed by a subsequent increase (Figure 1C,D). Further flow‐cytometric analysis of cardiac macrophage subsets, defined by CCR2 and MHC‐II expression levels [32, 33], showed that USP9X was significantly downregulated at day 3 post‐MI across multiple populations: embryonic‐origin resident macrophages (CCR2^−^ MHC‐II^high^ and CCR2^−^ MHC‐II^low^), newly recruited monocytes (CCR2^+^ MHC‐II^low^), and differentiated monocyte‐derived macrophages (CCR2^+^ MHC‐II^high^). These results suggest that USP9X reduction is a broad phenomenon affecting distinct macrophage subsets (Figure S1A,B). The downregulation was further confirmed by immunofluorescence staining, which revealed markedly lower USP9X expression in CD68^+^ cardiac macrophages at day 3 post‐MI compared with baseline (day 0), followed by a partial recovery by day 7 (Figure 1E,F). In parallel with the temporal changes in USP9X expression, Masson's trichrome staining of representative heart sections demonstrated the progression of myocardial injury across the indicated time point (Figure S1C). Next, to explore potential triggers of USP9X downregulation after MI, we stimulated macrophages in vitro with lipopolysaccharide (LPS) to activate Toll‐like receptor 4 (TLR4), a key innate immune pathway engaged by damage‐associated molecular patterns (DAMPs) released during myocardial injury [34, 35]. With prolonged LPS exposure, USP9X protein levels in macrophage progressively declined (Figure 1G,H). To further establish the physiological relevance of this observation, macrophages were treated with high mobility group box 1 (HMGB1), a prototypical DAMP released during myocardial injury [36, 37, 38]. HMGB1 stimulation similarly induced a time‐dependent reduction in USP9X protein expression (Figure S1D,E), supporting a role for DAMP‐driven inflammatory signaling in mediating USP9X downregulation following MI. Notably, *Usp9x* messenger RNA (mRNA) levels remained unchanged following LPS stimulation (Figure 1I). This suggests that the observed reduction in USP9X protein abundance is unlikely to be transcriptionally regulated, and may instead involve post‐translational mechanisms.

Collectively, these results reveal the dynamic regulation of USP9X in macrophages during early MI, suggesting its role in modulating cardiac inflammation.

### 2.2 USP9X Deficiency Exacerbates Cardiac Dysfunction Following MI in Mice

**FIGURE 2:**
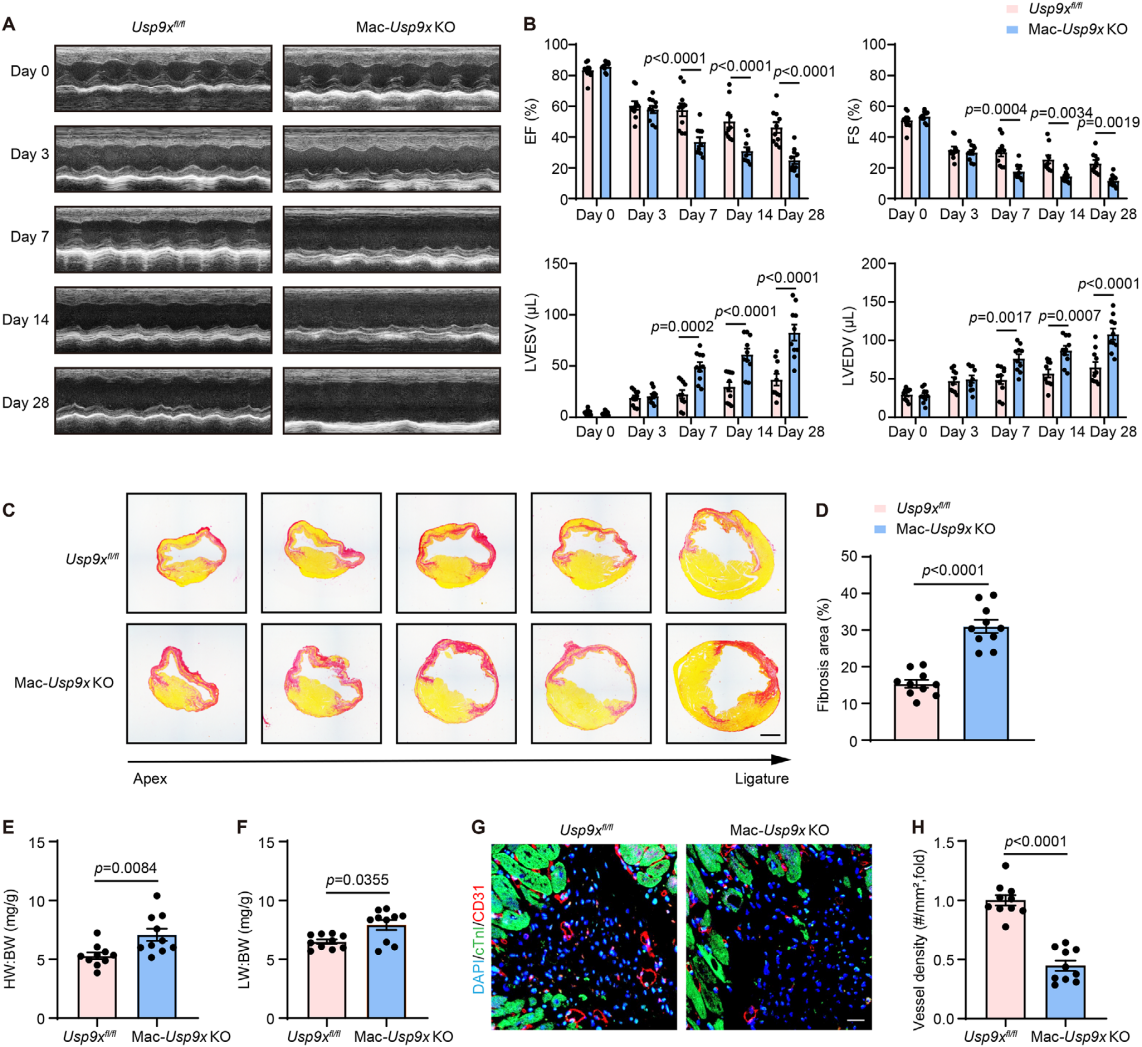
Macrophage USP9X deficiency aggravates cardiac dysfunction and impairs repair post‐MI in mice. MI surgery was performed in *Usp9x^fl/fl^* and Mac‐*Usp9x* KO mice, which were then assessed at baseline (day 0) and on days 3, 7, 14, and 28 post‐MI. (A) Representative M‐mode echocardiograms from the indicated groups at the specified time points. (B) Echocardiographic measurements of EF, FS, LVEDV, and LVESV. Two‐way ANOVA with Tukey's multiple comparisons test (*n* = 10). (C) Representative Sirius red‐stained cross‐sections of hearts (cut at 200 µm intervals) from the indicated groups at day 28 post‐MI. Scale bar, 1 mm. (D) Quantification of infarct size percentage from Panel C. Student's *t* test (*n* = 10). (E) HW:BW in MI‐operated mice, measured at 28 days post‐surgery. Student's *t* test (*n* = 10). (F) LW:BW in MI‐operated mice, measured at 28 days post surgery. Mann–Whitney *U*‐test (*n* = 10). (G) Representative immunofluorescence staining of CD31 (red), cardiac troponin I (cTnI, green), and DAPI (blue) in heart cross‐sections (border zone) from the indicated groups at day 28 post‐MI. Scale bar, 20 µm. (H) Quantitative analysis of microvessel density in border areas from G, Mann–Whitney *U*‐test (*n* = 10).

To elucidate the role of macrophage USP9X in myocardial repair following MI, we generated mice with macrophage‐specific deletion of *Usp9x* (Mac‐*Usp9x* knockout [KO]) by crossing *Usp9x^fl/fl^* mice with LysM‐Cre mice. The knockout efficiency was verified by genotypic PCR and further confirmed through WB analysis, showing markedly reduced USP9X protein levels in BMDMs from Mac‐*Usp9x* KO mice relative to *Usp9x^fl/fl^* littermate controls (Figure S2). Cardiac function was assessed post‐MI by echocardiography, measuring parameters including ejection fraction (EF), fractional shortening (FS), left ventricular end‐diastolic volume (LVEDV), and left ventricular end‐systolic volume (LVESV). Baseline measurements of these parameters were comparable between Mac‐*Usp9x* KO and littermate control mice prior to surgery and at 3 days post surgery. However, on days 7, 14, and 28 post‐MI, LVEDV and LVESV were significantly higher, and EF and FS were lower in Mac‐*Usp9x* KO mice than in *Usp9x^fl/fl^* controls (Figure 2A,B). Four weeks following MI, Mac‐*Usp9x* KO mice exhibited significantly larger fibrotic areas compared to control mice, as shown in Figure 2C,D. Furthermore, the ratios of heart weight‐to‐body weight (HW:BW) and lung weight‐to‐body weight (LW:BW) were increased in USP9X conditional knockout mice compared to controls (Figure 2E,F). Microvessel density in the infarct border zone at 28 days post‐MI was also significantly lower in Mac‐*Usp9x* KO mice than in controls (Figure 2G,H). In parallel, to investigate the impact of USP9X inhibition, mice were treated with the USP9X inhibitor WP1130 twice weekly, commencing 7 days prior to MI surgery and continuing for 28 days post‐MI. This pharmacological intervention also led to significant adverse outcomes, characterized by impaired cardiac function (elevated LVEDV and LVESV, reduced EF and FS) and increased HW:BW and LW:BW ratios. Additionally, histological analysis revealed an enlarged scar size and reduced angiogenesis in WP1130‐treated mice compared to vehicle‐treated controls (Figure S3). Collectively, these findings suggest that macrophage‐specific USP9X deficiency impedes the cardiac repair following MI.

### 2.3 Macrophage USP9X Ablation Aggravates Post‐MI Inflammation

**FIGURE 3:**
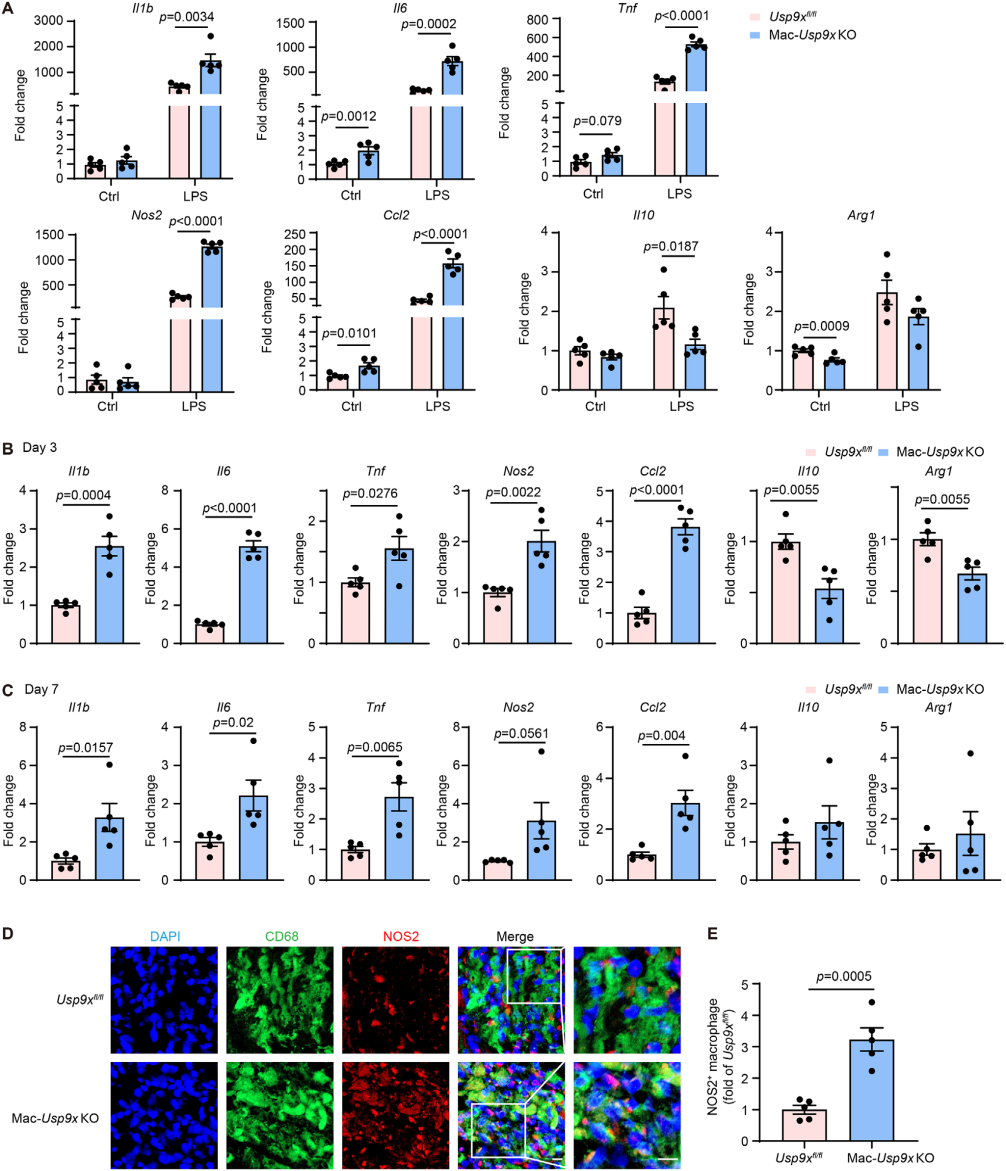
Knockout of USP9X in macrophages promotes a pro‐inflammatory phenotype. (A) Quantitative PCR analysis of indicated gene expressions in BMDMs isolated from *Usp9x^fl/fl^* and Mac‐*Usp9x* KO mice and treated with PBS or LPS (100 ng/mL) for 24 h. Target gene expression was normalized to *Actb* mRNA levels. Two‐way ANOVA with Tukey's multiple comparisons test (*n* = 5). (B,C) Quantitative PCR analysis of indicated genes in heart tissues from *Usp9x^fl/fl^* and Mac‐*Usp9x* KO mice at (B) day 3 and (C) day 7 post‐MI. Target gene expression was normalized to *Actb* mRNA levels. Student's *t* test (*n* = 5). (D) Representative immunofluorescence staining of NOS2 (red), CD68 (green), and DAPI (blue) in heart cryosections (border zone) from the indicated groups at day 3 post‐MI. Scale bar, 20 µm. (E) Quantitative analysis of NOS2 positive macrophages in border areas of hearts from Panel D. Student's *t* test (*n* = 5).

To further investigate the role of macrophage USP9X in post‐MI inflammation, we isolated BMDMs from Mac‐*Usp9x* KO mice and their littermate controls. Upon LPS stimulation in vitro, USP9X‐deficient BMDMs exhibited higher expression of pro‐inflammatory genes (*Il1b, Il6, Tnf, Nos2*, and *Ccl2*) and lower expression of anti‐inflammatory markers (*Il10* and *Arg1*) compared to controls (Figure 3A). Similar trends were observed in BMDMs from WT mice treated with the USP9X inhibitor WP1130 (Figure S4A).

In vivo, the mRNA levels of pro‐inflammatory genes were significantly higher in the infarct regions of Mac‐*Usp9x* KO mice compared to *Usp9x^fl/fl^* controls at 3 and 7 days post‐MI (Figure 3B,C). This trend was also observed in WP1130‐treated mice compared to controls at 3 and 7 days post‐MI (Figure S4B,C). Consistent with these findings, immunofluorescence analysis showed significantly higher NOS2 expression within CD68^+^ areas of heart sections from USP9X‐deficient or WP1130‐treated mice compared to controls (Figure 3D,E and Figure S4D,E). NOS2 is a well‐established marker for a functionally polarized, pro‐inflammatory, and tissue‐destructive macrophage phenotype in pathological tissue remodeling [39, 40].

Collectively, these results suggest that USP9X inhibition promotes a pro‐inflammatory macrophage phenotype and impairs inflammation resolution in the heart following MI.

### 2.4 USP9X Suppresses Inflammatory Responses in Macrophages by Deubiquitinating and Stabilizing TRAF‐Type Zinc Finger Domain Containing 1

Our previous studies demonstrated that the inhibition of USP9X promotes K63‐linked ubiquitination at lysine 27 (K27) of SR‐A1, leading to its internalization from the cell surface. This process enhances foam cell formation and cytokine production, thereby exacerbating inflammatory responses in atherosclerosis [23]. Given these findings, we hypothesized that this mechanism may contribute to increased inflammation following MI. To investigate this, we generated RAW264.7 cells stably expressing either SR‐A1^WT^ or SR‐A1^K27R^ fusion proteins via lentiviral transduction. Consistent with our previous findings, the K27R mutation significantly attenuated inflammatory gene expressions compared to cells expressing SR‐A1^WT^. However, in macrophages expressing SR‐A1^K27R^, USP9X deficiency still upregulated inflammatory genes (Figure S5A). These observations suggest the existence of an additional, SR‐A1‐independent mechanism by which USP9X regulates inflammatory responses in macrophages.

**FIGURE 4:**
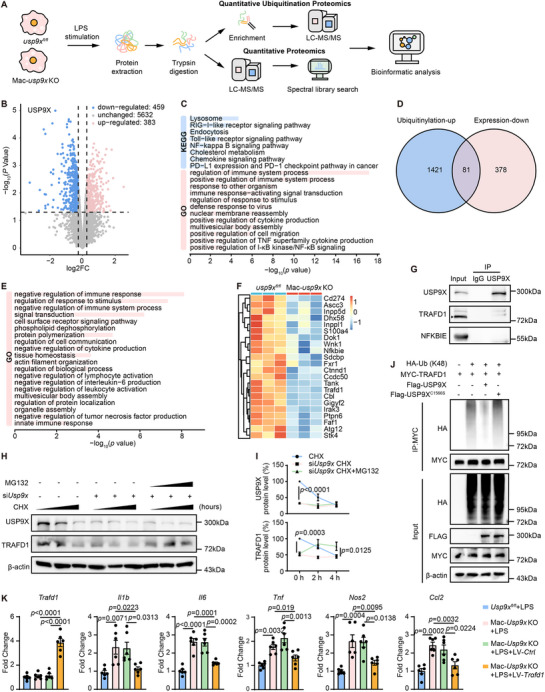
USP9X deubiquitinates and stabilizes TRAFD1 to regulate macrophage‐mediated inflammatory responses. (A) Workflow for quantitative ubiquitinome and proteome profiling in BMDMs from *usp9x^fl/fl^* and Mac‐*usp9x* KO mice stimulated with LPS for 12 h. (B) Volcano plot illustrating differences in protein expression between Mac‐*usp9x* KO and control BMDMs. (C) KEGG and GO enrichment analyses of differentially expressed proteins. (D) Venn diagrams showing overlap between proteins exhibiting increased ubiquitination (blue) and proteins with downregulated expression (pink) in USP9X‐deficient BMDMs. (E) GO enrichment analysis of the 81 overlapping candidate proteins identified in Panel D. (F) Heatmap of candidate proteins based on their role in negative regulation of immune response. (G) whole cell lysates (WCL) from BMDMs were subjected to IP with anti‐USP9X or control rabbit immunoglobulin G (IgG) antibody, followed by immunoblotting with antibodies against the indicated proteins (*n* = 3). (H) After being transfected with or without siUsp9x for 48 h, BMDMs were primed with LPS for 12 h and then incubated with CHX (50 µg/mL) or MG132 (10 µm) for the indicated times (0, 2, and 4 h). Endogenous levels of TRAFD1 and USP9X were assessed by WB analysis. (I) Quantification of TRAFD1 protein turnover rate from Panel H. Protein levels were normalized to levels at time zero (0 h) in the control group. Two‐way ANOVA with Tukey's multiple comparisons test (*n* = 3). (J) HEK293 cells were co‐transfected with HA‐Ub, Myc‐tagged TRAFD1 (Myc–TRAFD1)MYC–TRAFD1, rtTA vectors, and either FLAG‐tagged wild‐type USP9X or a catalytically inactive USP9X mutant (C1566S). After 24 h, protein expression was induced with doxycycline (DOX, 1 µg/mL) for an additional 24 h. Cells were treated with MG132 for the final 2 h before harvest. MYC–TRAFD1 was immunoprecipitated using MYC magnetic beads and analyzed by immunoblotting with the indicated antibodies (*n* = 3). (K) BMDMs from *Usp9x^fl/fl^* and Mac‐*Usp9x* KO mice were transduced with lentiviral vectors expressing either a control sequence (LV‐*Ctrl*) or TRAFD1 (LV‐*Trafd1*) for 48 h, followed by a 24 h stimulation with PBS or LPS. mRNA levels of the indicated genes were quantified by qPCR and normalized to *Actb*. One‐way ANOVA with Tukey's multiple comparisons test (*n* = 5).

To identify potential USP9X substrates involved in this alternative pathway, we performed global proteomic and ubiquitinomic profiling in LPS‐stimulated BMDMs from Mac‐*usp9x* KO and *usp9x^fl/fl^* control mice (Figure 4A). Proteomic analysis identified 459 downregulated and 383 upregulated proteins in USP9X^−^deficient BMDMs compared to controls. Importantly, USP9X itself was among the significantly downregulated proteins, confirming both the efficacy of the genetic deletion and the reliability of the dataset (Figure 4B). Kyoto Encyclopedia of Genes and Genomes (KEGG) and Gene Ontology (GO) enrichment analyses of these proteomic changes revealed significant enrichment for proteins involved in inflammatory signaling pathways, including TLR and nuclear factor kappa B (NF ‐ κB) signaling. Specifically, the differentially expressioned proteins were enriched in terms related to immune activation, such as “positive regulation of immune system process” and “positive regulation of I‐κB kinase/NF‐κB signaling” (Figure 4C). This enrichment pattern, together with the upregulation of pro‐inflammatory proteins and downregulation of inflammation‐resolving proteins observed in the heat map (Figure S5B), corroborates our prior phenotypic observation that USP9X deficiency enhances inflammatory gene expression.

Since increased ubiquitination typically promotes degradation, we focused on proteins that were both downregulated at the expression level and exhibited increased ubiquitination in USP9X‐deficient macrophages. Ubiquitinomic analysis identified 1421 proteins with significantly increased ubiquitination. Cross‐referencing this list with the 378 significantly downregulated proteins identified in the proteomic analysis yielded 81 candidate proteins (Figure 4D). These candidates represent high‐confidence targets whose reduced abundance is likely attributable to enhanced ubiquitination following USP9X ablation. GO enrichment analysis of these 81 proteins showed significant enrichment in biological processes such as “negative regulation of immune response” and “negative regulation of cytokine production” (Figure 4E), consistent with the hyper‐inflammatory phenotype observed in USP9X‐deficient macrophages. Based on these findings, we prioritized negative regulators of inflammatory signaling from the candidate list, as depicted in the heatmap (Figure 4F). Among these, NF‐κB inhibitor epsilon (NFKBIE) showed the most significant decrease in protein expression, while TRAF‐type zinc finger domain containing 1 (TRAFD1) exhibited the most significant increase in ubiquitination. To determine direct interactions, we performed immunoprecipitation (IP) assays and found that USP9X specifically interacts with TRAFD1, but not with NFKBIE (Figure 4G). This specific physical interaction, combined with the increased ubiquitination and decreased protein levels of TRAFD1 upon USP9X deletion, strongly suggests that TRAFD1 is a direct substrate of USP9X in macrophages. TRAFD1 functions as a negative feedback regulator in the TLR signaling pathway, controlling excessive immune responses [41, 42, 43]. To further investigate the regulatory role of USP9X in TRAFD1 stability, we evaluated TRAFD1 protein turnover using cycloheximide (CHX) chase assays. In LPS‐pretreated macrophages, USP9X knockdown or pharmacological inhibition significantly accelerated TRAFD1 degradation in macrophages, which was blocked by the proteasome inhibitor MG132 (Figure 4H,I and Figure S5C,D), indicating that USP9X stabilizes TRAFD1 by preventing proteasomal degradation. Consistently, pharmacological inhibition of USP9X with WP1130 increased the overall ubiquitination of TRAFD1 (Figure S5E). To further define the specific mechanism of this regulation, we sought to identify the ubiquitin chain linkage targeted by USP9X. Given that the catalytic domain of USP9X preferentially acts on K11‐linked, followed by K63‐ and K48‐linked polyubiquitin chains [44], and that both K11‐ and K48‐linked chains can mediate proteasomal degradation [45, 46], we performed additional IP analysis to distinguish between these possibilities. Our results revealed that USP9X specifically removes K48‐linked ubiquitin from TRAFD1, rather than K11‐linked chains, thereby stabilizing the protein (Figure S5F). To verify that TRAFD1 is a direct target of USP9X, we used a catalytically inactive mutant (USP9X^C1566S^). Importantly, this mutant failed to deubiquitinate TRAFD1, resulting in its enhanced ubiquitination (Figure 4J). This provides critical evidence that TRAFD1 stability is governed by the enzymatic activity of USP9X.

To further validate the anti‐inflammatory role of TRAFD1 in macrophages, we performed loss‐of‐function studies. Knockdown of TRAFD1 significantly upregulated the expression of inflammatory genes in macrophages (Figure S5G). Following LPS treatment, the pro‐inflammatory phenotype resulting from TRAFD1 deficiency mirrored that observed upon USP9X deletion, suggesting their involvement in a common regulatory pathway. To determine whether TRAFD1 acts as a critical downstream effector mediating the anti‐inflammatory function of USP9X, we conducted a rescue experiment. Lentivirus‐mediated overexpression of TRAFD1 in USP9X‐knockout macrophages reversed the upregulation of pro‐inflammatory genes induced by USP9X deficiency (Figure 4K). These results indicate that TRAFD1 is a key substrate through which USP9X regulates inflammatory responses in macrophages.

Together, these data demonstrate that USP9X deubiquitinase activity stabilizes TRAFD1 by removing K48‐linked polyubiquitin chains, which prevents TRAFD1 from proteasomal degradation and subsequently suppresses the inflammatory response in macrophages.

### 2.5 USP9X Degradation by CMA in Inflammatory Macrophages

**FIGURE 5:**
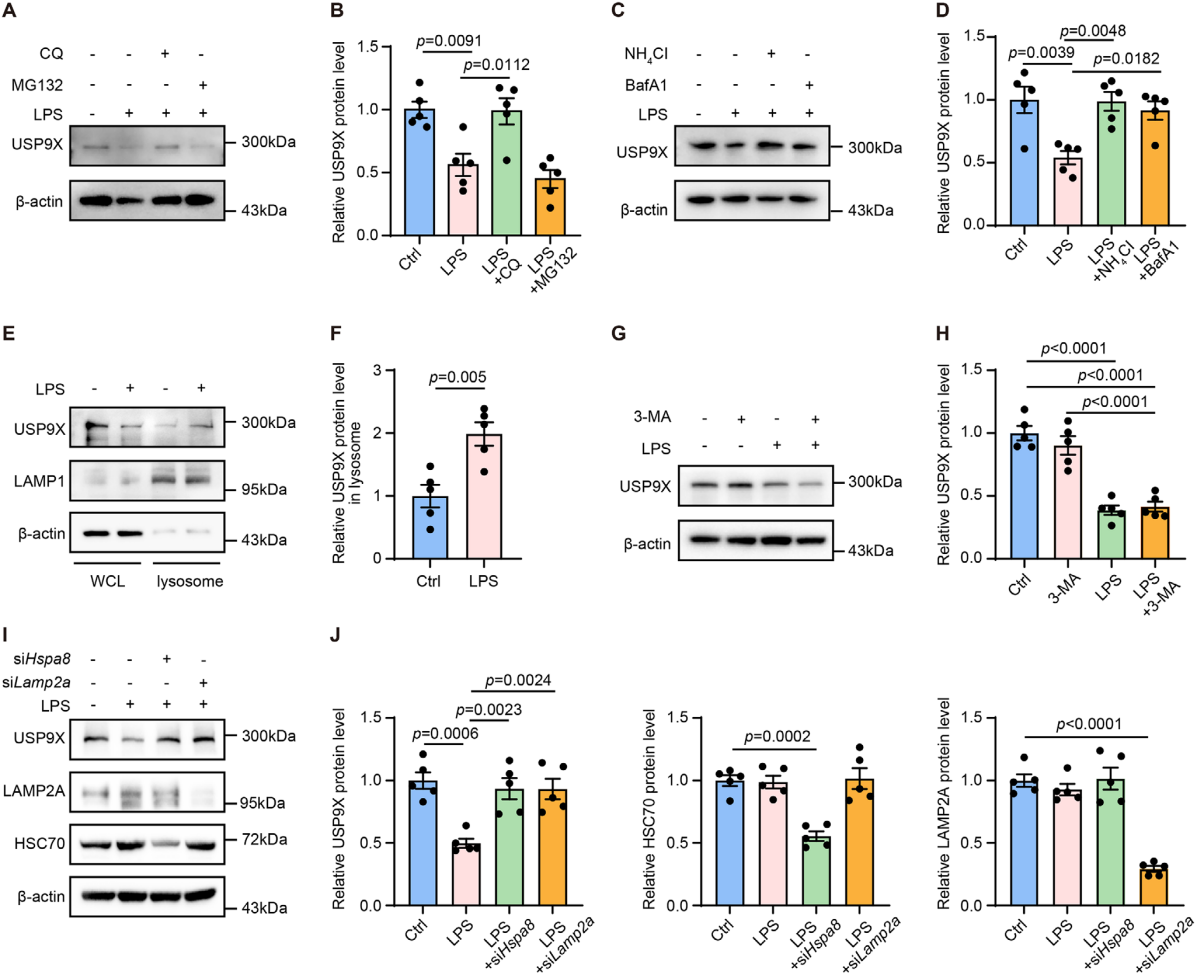
CMA mediates USP9X degradation in macrophages during inflammation. (A) BMDMs were pretreated with LPS (100 ng/mL) for 24 h, then treated with CQ or MG132 as indicated, cell lysates were analyzed by WB. (B) Quantification of USP9X protein expression from Panel A. One‐way ANOVA with Tukey's multiple comparisons test (*n* = 5). (C) BMDMs were treated with LPS, BafA1 (100 nm), or NH_4_Cl (10 mm) for 24 h as indicated. Cell lysates were collected for immunoblot analysis. (D) Quantification of USP9X protein levels from Panel C, one‐way ANOVA with Tukey's multiple comparisons test (*n* = 5). (E) WB analysis of USP9X in WCL and lysosome‐enriched fractions from BMDMs treated with or without LPS for 12 h. LAMP1 and β‐actin served as lysosomal and loading controls, respectively. (F) Quantification of USP9X levels in the lysosome fraction normalized to LAMP1; Student's *t* test (*n* = 5). (G) BMDMs were treated with vehicle, LPS or 3‐MA (5 mm) for 24 h as indicated. Cell lysates were analyzed by WB. (H) Quantification of USP9X protein levels from Panel G, one‐way ANOVA with Tukey's multiple comparisons test (*n* = 5). (I) BMDMs were transfected with si*Ctrl*, si*Hspa8* or si*Lamp2a* for 48 h, then stimulated with LPS (100 ng/mL) or vehicle for 24 h. Cell lysates were analyzed by WB. (J) Quantification of USP9X, HSC70 and LAMP2A protein levels from Panel I. One‐way ANOVA with Tukey's multiple comparisons test (*n* = 5).

Having established that USP9X restrains inflammatory signaling by stabilizing TRAFD1 through deubiquitination, we next sought to understand how USP9X itself is regulated during macrophage activation. Given that USP9X protein levels were markedly reduced under inflammatory conditions, while its mRNA levels remained unchanged, we hypothesized that post‐translational mechanisms, particularly protein degradation, may account for this downregulation. To explore this, we investigated the contribution of the two major intracellular protein degradation pathways, namely the ubiquitin‐proteasome system and the lysosomal pathway, in controlling USP9X stability in macrophages [25]. We first performed pharmacological inhibition experiments by administering either proteasomal or lysosomal inhibitors. Lysosomal inhibition with chloroquine (CQ) effectively reversed LPS‐induced USP9X downregulation, whereas proteasomal inhibition with MG132 had no significant effect (Figure 5A,B). These results indicate that USP9X degradation during inflammation is primarily mediated through the lysosomal pathway. The lysosomal dependency was further corroborated by parallel experiments using alternative lysosomal inhibitors, including bafilomycin A1 (BafA1) and NH_4_Cl, which similarly rescued USP9X expression following LPS stimulation (Figure 5C,D). To further validate the lysosomal localization of USP9X during inflammation, we performed lysosomal isolation assays. The results demonstrated a significant enrichment of USP9X in the lysosomal fraction upon LPS stimulation (Figure 5E,F). These results collectively demonstrate that inflammatory stimuli induce USP9X degradation primarily through the lysosomal pathway.

Given the predominant cytoplasmic localization of USP9X, we focused our investigation on autophagic‐lysosomal degradation mechanisms rather than endocytic or phagocytic pathways. Notably, inhibition of macroautophagy using 5 mm 3‐methyladenine (3‐MA), a class III phosphatidylinositol 3‐kinase inhibitor [47], did not significantly affect LPS‐induced USP9X downregulation (Figure 5G,H), thereby excluding conventional macroautophagy as the primary degradation mechanism. We subsequently explored CMA, which specifically degrades target proteins via direct lysosomal translocation in a non‐vesicle‐dependent manner [27, 48]. We performed genetic knockdown of key mediators of this pathway: HSC70 and LAMP2A. HSC70, encoded by *Hspa8*, serves as the canonical chaperone for CMA substrate recognition, whereas LAMP2A constitutes the lysosomal translocation complex [49, 50]. Knockdown of either HSC70 or LAMP2A substantially restored USP9X protein levels in LPS‐challenged macrophages (Figure 5I,J). Collectively, these data identify CMA as the specific lysosomal pathway responsible for USP9X degradation ininflammatory macrophages.

### 2.6 Acetylation‐Enhanced Interaction With HSC70 Mediates CMA‐Dependent Degradation of USP9X in Inflammatory Macrophages

**FIGURE 6:**
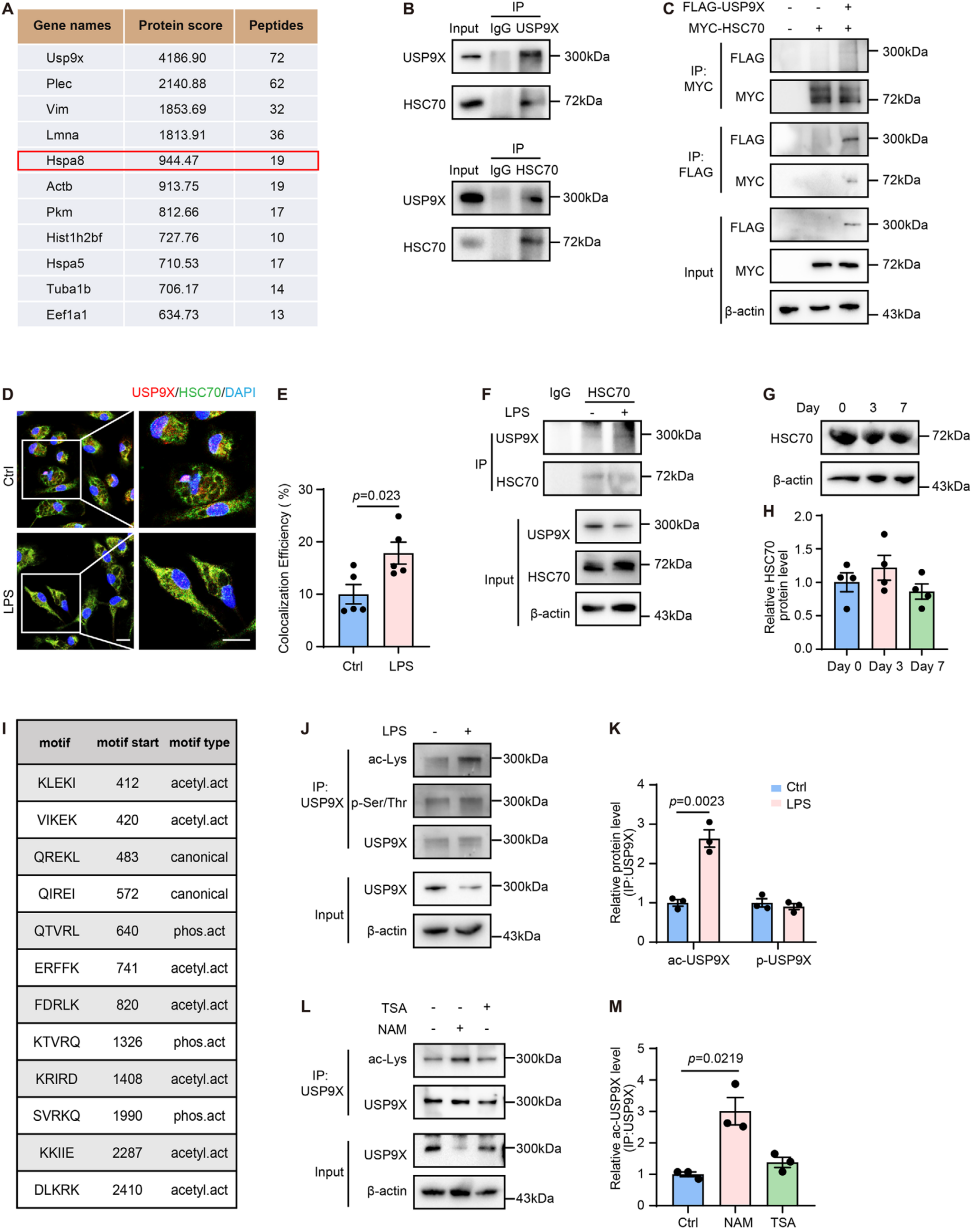
Increased acetylation of USP9X in inflammatory macrophages facilitates its degradation via HSC70‐mediated CMA. (A) Representative mass spectrometry data identified HSPA8 (HSC70) as a USP9X‐interacting protein; interaction score and number of unique peptides are shown. (B) WCL were subjected to IP using antibodies against USP9X, HSC70, or control rabbit IgG. The immunoprecipitates were then analyzed by immunoblotting with anti‐HSC70 or anti‐USP9X antibodies, as indicated (*n* = 3). (C) Co‐IP analysis of the association between USP9X and HSC70 in HEK293 cells co‐transfected with FLAG‐USP9X, rtTA vectors, and MYC‐HSC70 for 24 h. Expression was induced with DOX (1 µg/mL) for an additional 24 h before analysis (*n* = 3). (D) Immunofluorescence staining of USP9X (red) and HSC70 (green) in macrophages treated with vehicle or LPS for 24 h. Scale bar, 10 µm. (E) Quantification of USP9X and HSC70 colocalization shown in Panel D. Student's *t* test (*n* = 5). (F) BMDMs were treated with vehicle or LPS for 24 h, followed by IP with anti‐HSC70 or rabbit IgG antibody and then immunoblotted with indicated antibodies (*n* = 3). (G) WB analysis of HSC70 expression in cardiac macrophages isolated from WT mice at indicated time points after MI. (H) Quantification of HSC70 protein expression in Panel G. One‐way ANOVA with Tukey's multiple comparisons test (*n* = 4). (I) Schematic representation of predicted KFERQ‐like motifs within the USP9X protein sequence; motif sequence and motif type (intrinsic, acetylation‐dependent latent, phosphorylation‐dependent latent) are indicated. (J) BMDMs were treated with vehicle or LPS for 24 h, followed by IP with anti‐USP9X antibody and then immunoblotted with indicated antibody. (K) Quantification of USP9X acetylation and phosphorylation levels, normalized to USP9X in indicated immunoprecipitates. Student's *t* test (*n* = 3). (L) BMDMs were treated with vehicle, TSA (100 nm) or NAM (5 mm) for 24 h, followed by IP with anti‐USP9X antibody and then immunoblotted with indicated antibody. (M) Quantification of USP9X acetylation and phosphorylation levels in Panel L, normalized to total USP9X levels in indicated immunoprecipitates. Kruskal–Wallis test with Dunn's multiple comparisons test (*n* = 3).

To further elucidate the underlying mechanisms of HSC70‐mediated CMA in the degradation of USP9X in macrophages under inflammatory conditions, we performed IP of macrophage lysates using an anti‐USP9X antibody. Subsequent liquid chromatography–tandem mass spectrometry (LC – MS/MS) analysis of the precipitated complexes identified HSC70 (Figure 6A), providing initial evidence for the interaction between USP9X and HSC70 in macrophages.

To validate this interaction, endogenous co‐immunoprecipitation (co‐IP) experiments were conducted in BMDMs. Total protein lysates were subjected to IP using antibodies against endogenous USP9X or HSC70, and the results demonstrated an interaction between the two proteins (Figure 6B). Moreover, FLAG‐tagged USP9X and MYC‐tagged HSC70 plasmids were co‐transfected into HEK293 cells, confirming the exogenous interaction between USP9X and HSC70 (Figure 6C). For domain mapping, four truncated FLAG‐tagged USP9X variants were generated: the N‐terminus containing the α–α supercoil domain (N), the middle region of USP9X (M), the segment containing the catalytic ubiquitin‐specific protease domain (C1) and C‐terminal regulatory domain (C2) (Figure S6A). Co‐expression of these fragments with MYC‐HSC70 in HEK293 cells revealed selective interaction between the C2 domain of USP9X and HSC70, while no binding was observed with N, M, or C1 domains (Figure S6B). Importantly, the interaction between USP9X and HSC70 is enhanced in an inflammation‐dependent manner. Immunofluorescence analysis showed that LPS stimulation markedly alters their subcellular distribution, shifting from a diffuse cytoplasmic pattern with minimal overlap under basal conditions to pronounced co‐localization within distinct puncta (yellow in merged images; Figure 6D,E). This observed increased in proximity was further confirmed by IP assays (Figure 6F). Collectively, these findings suggest that USP9X downregulation during macrophage inflammation is driven by CMA and mediated via increased USP9X‐HSC70 interaction, which targets USP9X for lysosomal degradation.

To further investigate the regulatory axis controlling HSC70‐dependent USP9X degradation during inflammation, we assessed the expression dynamics of HSC70 in cardiac macrophages isolated from MI mouse models, as well as in macrophages stimulated with LPS in vitro. Immunoblot quantification demonstrated stable HSC70 protein levels across conditions (Figure 6G,H and Figure S6C,D), indicating that USP9X degradation is regulated through functional activation of HSC70 rather than modulation of its expression level.

The molecular recognition mechanism of CMA substrates centers on KFERQ‐like motifs, which are defined by four conserved features: flanking glutamine (*Q*) residues, at least one positively charged residue (K/R), at least one hydrophobic residue (L/I/V/F), and at least one acidic residue (E/D). Importantly, post‐translational modifications can dynamically generate latent KFERQ‐like motifs through mechanisms such as charge mimicry (phosphorylation of serine, threonine, or tyrosine introduces negative charges), similar properties (lysine acetylation mimics the side chain of glutamine) and conformational switching (ubiquitination or palmitoylation alters the tertiary structure of the protein) [26, 51, 52].

Using the KFERQ Finder software v0.8 [53], we identified two intrinsic KFERQ motifs, seven acetylation‐dependent latent motifs (Q‐mimic via lysine acetylation) and three phosphorylation‐dependent latent motifs within USP9X (Figure 6I). Consistent with these predictions, IP assays revealed that USP9X undergoes increased acetylation following inflammatory stimulation (Figure 6J,K). These results indicated that the acetylation of USP9X in macrophages during post‐MI inflammation may enhance the exposure of KFERQ motifs, thereby promoting the autophagic degradation of USP9X mediated by HSC70.

To further elucidate the regulatory mechanisms governing USP9X acetylation dynamics in macrophages under inflammatory conditions, we implemented pharmacological modulation of cellular acetylation pathways. Administration of trichostatin A (TSA), a potent class I/II histone deacetylase inhibitor [54], failed to induce significant alterations in USP9X acetylation status. In contrast, pharmacological inhibition of sirtuin deacetylases using nicotinamide (NAM) provoked a marked elevation in USP9X acetylation levels (Figure 6L,M). Our observation that USP9X acetylation increases under inflammatory conditions is consistent with the established paradigm of SIRT1 suppression [55, 56, 57]. This increase in acetylation may represent a novel, downstream component of the inflammatory amplification cascade.

### 2.7 Peptide‐Mediated Competitive Inhibition of HSC70–USP9X Interaction Attenuates Macrophage‐Driven Inflammation Post‐MI

Based on the predicted acetylation‐prone sequences in USP9X that generate KFERQ‐like motifs, we designed seven synthetic peptides mimicking these KFERQ‐like motifs (Table S1). These peptides were engineered as competitive inhibitors to interfere with HSC70 recognition. To enhance cellular permeability, the peptides were structurally optimized through N‐terminal conjugation with the cell‐penetrating peptide (YGRKKRRQRRR).

**FIGURE 7:**
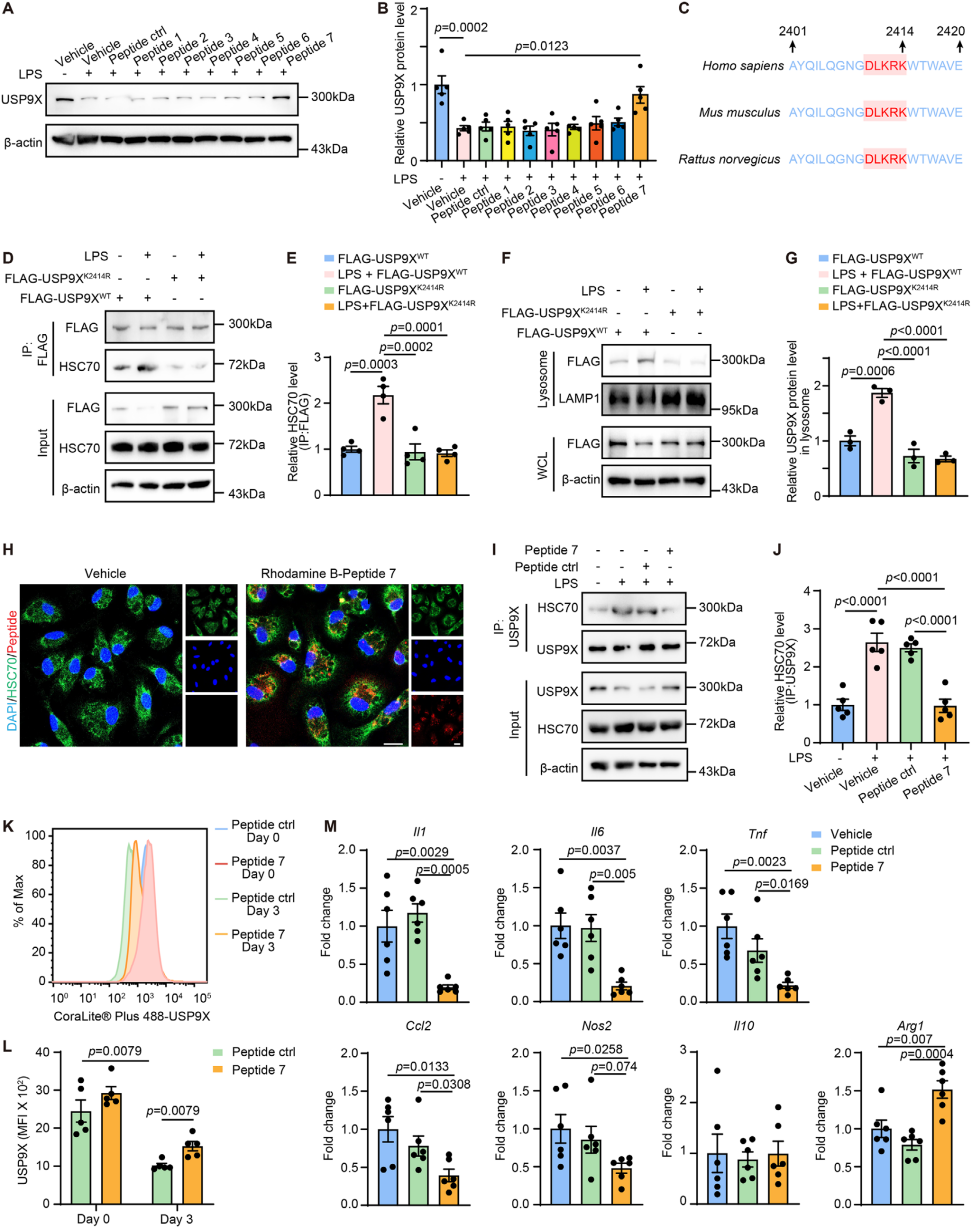
A peptide inhibits HSC70‐mediated USP9X degradation in macrophages to promote inflammation resolution following MI. (A) BMDMs were treated with vehicle or indicated peptides (20 µm) for 24 h. Cell lysates were analyzed by WB for USP9X expression. (B) Quantification of USP9X protein expression shown in Panel A. One‐way ANOVA with Tukey's multiple comparisons test (*n* = 5). (C) Multiple sequence alignment of USP9X region containing the critical motif across species, including human (*Homo sapiens*), mouse (*Mus musculus*) and rat (*Rattus norvegicus*). (D) BMDMs were transduced with lentiviral vectors encoding lentiviral vector FLAG‐USP9X^wt^ or USP9X^K2414R^ for 48 h, then stimulated with PBS or LPS for an additional 24 h. Lysates were subjected to anti‐FLAG IP; co‐precipitated HSC70 was detected by immunoblot. (E) Quantification of relative HSC70 levels bound to USP9X, normalized to immunoprecipitated FLAG levels. One‐way ANOVA with Tukey's multiple comparisons test (*n* = 4). (F) WCL and lysosome‐enriched fractions were prepared from BMDMs of the indicated treatment groups; immunoblots were probed for USP9X. LAMP1 served as lysosomal controls. (G) Quantification of USP9X levels in the lysosome fraction normalized to LAMP1. One‐way ANOVA with Tukey's multiple comparisons test (*n* = 3). (H) BMDMs were treated with vehicle or Rhodamine B–peptide 7 (20 µm) for 2 h, followed by immunofluorescence staining of HSC70 (green). Scale bar, 10 µm. (I) BMDMs were treated with LPS and indicated peptides for 24 h, Cell lysates were subjected to IP with anti‐USP9X antibody to assess the interaction between USP9X and HSC70. (J) Quantification of relative HSC70 levels bound to USP9X, normalized to immunoprecipitated USP9X levels. One‐way ANOVA with Tukey's multiple comparisons test (*n* = 5). (K) Flow‐cytometry analysis of USP9X expression in cardiac macrophages isolated from WT mice treated with vehicle or indicated peptides (20 mg/kg/d, 100 µL, intraperitoneal injection, administered starting at day 0 and continuing every 2 days) at 3 days post‐MI or sham surgery. (L) Quantification of USP9X MFI shown in Panel K. Mann–Whitney *U*‐test (*n* = 5). (M) Quantitative PCR analysis of indicated gene expression in heart tissues from vehicle‐ or indicated peptide‐treated mice (20 mg/kg/d, 100 µL, intraperitoneal injection) at day 3 post‐MI. Target gene expression was normalized to *Actb* mRNA levels. One‐way ANOVA with Tukey's multiple comparisons test (*n* = 6).

In LPS‐stimulated BMDMs, peptide 7 (YGRKKRRQRRR‐DLKRQ) specifically inhibited the degradation of USP9X, whereas the other peptides did not (Figure 7A,B). This finding suggests that lysine 2414 acetylation generates a functional KFERQ‐like motif recognized by HSC70 during inflammatory activation. Cross‐species evolutionary conservation of the DLKRK motif in USP9X highlighted its critical functional significance, supporting the broad therapeutic potential of our acetylated lysine‐mimetic peptide across diverse species (Figure 7C).

To further validate the role of K2414 in regulating USP9X stability, BMDMs were transduced with lentiviruses expressing FLAG‐USP9X^WT^ or the acetyl‐mimetic‐defective mutant FLAG‐USP9X^K2414R^. CHX chase assays demonstrated that FLAG‐USP9X^K2414R^ exhibited significantly delayed degradation compared with FLAG‐USP9X^WT^ upon LPS stimulation (Figure S7A,B). Moreover, unlike FLAG‐USP9X^WT^, the K2414R mutant failed to enhance its interaction with HSC70 in response to LPS stimulation (Figure 7D,E). Consistently, FLAG‐USP9X^K2414R^ did not increase its lysosomal localization after LPS treatment, in contrast to the pronounced lysosomal accumulation observed for the WT protein (Figure 7F,G).

Immunofluorescence imaging confirmed intracellular delivery of Rhodamine B–tagged peptide 7 and its co‐localization with HSC70 in macrophages (Figure 7H). Next, we pretreated BMDMs with LPS and then treated them with peptide 7 or scrambled control peptide (peptide ctrl: YGRKKRRQRRR‐RDKLQ). IP assays revealed that peptide 7, rather than with the control peptide, significantly attenuated HSC70–USP9X interactions and stabilized USP9X protein levels under inflammatory challenge (Figure 7I,J).

To assess the in vivo efficacy of peptide 7, we isolated cardiac macrophages from MI mice and sham‐operated controls. Each group was treated with either peptide 7 or peptide ctrl. Flow‐cytometry analysis revealed that peptide 7 treatment effectively attenuated the MI‐induced downregulation of USP9X in macrophages at 3 days post‐MI, as evidenced by fluorescence intensity measurements (Figure 7K,L and Figure S7C). Notably, quantitative PCR profiling of infarcted myocardium demonstrated reduced expression of inflammatory genes (*Il1b*, *Il6*, *Tnf*, *Nos2*, and *Ccl2*) and elevated expression of anti‐inflammatory gene *Arg1* in peptide 7‐treated mice compared to both peptide ctrl‐ and vehicle‐treated groups. No observable therapeutic difference was found between the peptide ctrl and vehicle groups (Figure 7M). Furthermore, a dose–response study incorporating a higher‐dose group revealed that the anti‐inflammatory effect of peptide 7 was partially dose dependent (Figure S7D). While the higher dose led to a further reduction in the expression of cardiac inflammatory genes *Il6* and *Ccl2* compared to the lower dose, it did not significantly enhance the suppression of other key markers, including *Il1b*, *Tnf*, and *Nos2*. Based on its optimal efficacy‐to‐safety profile and the absence of significant additional therapeutic benefit at higher concentrations in preliminary assessments, the lower dose was selected for subsequent studies. This anti‐inflammatory phenotype was replicated in vitro using LPS‐primed BMDMs, where peptide 7 specifically downregulated pro‐inflammatory genes and upregulated *Arg1* (Figure S7E).

To investigate the cell‐specific effects of peptides, we analyzed their influence on cardiomyocytes and cardiac fibroblasts using key molecular markers. Treatment with peptide 7 did not significantly alter the expression of hypertrophy markers (ANP and Myh7) or USP9X in cardiomyocytes (Figure S8A,B). Similarly, in cardiac fibroblasts, neither peptide ctrl nor peptide 7 affected the levels of fibrosis markers (fibronectin and alpha–smooth muscle actin [α ‐ SMA]) or USP9X (Figure S8C,D). These findings indicate that peptide 7 does not directly modulate hypertrophic or fibrotic pathways in these cell types, supporting its macrophage‐specific action. Additionally, methylthiazolyldiphenyl ‐ tetrazolium bromide (MTT) assay revealed no significant cytotoxicity in cardiomyocytes following prolonged exposure to either peptide (Figure S8E), indicating its potential safety for cardiac applications.

To further examine whether peptide 7 broadly affects CMA, we evaluated protein levels of known CMA substrates, including CHK1 and GPX4 [58, 59]. Peptide 7 did not alter the expression of these substrates either under basal or LPS‐stimulated conditions (Figure S8F,G), confirming that its stabilizing effect is selective for USP9X and does not apply to unrelated CMA targets. Collectively, these results demonstrate that peptide 7 effectively inhibits HSC70‐mediated USP9X degradation, attenuates macrophage‐driven inflammation post‐MI in a dose‐responsive manner, and exhibits cellular and substrate specificity. These findings support its therapeutic potential for mitigating inflammatory conditions following MI.

### 2.8 An Inhibitory Peptide That Targeting the HSC70–USP9X Interaction Promotes Cardiac Repair Post‐MI

**FIGURE 8:**
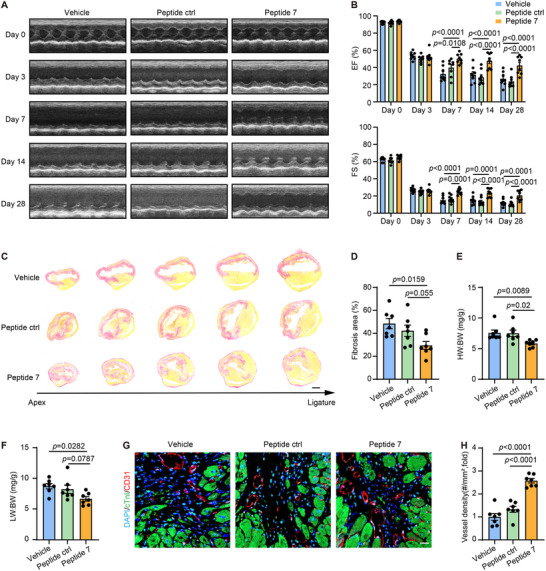
Treatment with peptide 7 improved cardiac repair in mice post‐MI. WT mice subjected to MI surgery were administrated with vehicle or indicated peptides (20 mg/kg, 100 µL, three times a week, intraperitoneal injection) for 28 days. (A) Representative M‐mode echocardiograms obtained on days 0, 3, 7, 14, and 28 after MI from mice in indicated groups. (B) Echocardiographic measurements of EF and FS in indicated groups on days 0, 3, 7, 14, and 28 post‐MI. Two‐way ANOVA with Tukey multiple comparisons test (*n* = 7). (C) Sirius red staining of sequential heart cross‐sections from each block were cut at 200 µm intervals in indicated groups on day 28 after MI, scale bar, 1 mm. (D) Quantification of percentage of infarct size in Panel C. One‐way ANOVA with Tukey multiple comparisons test (*n* = 7). (E) HW:BW in MI‐operated mice, measured 28 days post surgery. Kruskal–Wallis test with Dunn's multiple comparisons test (*n* = 7). (F) LW:BW in MI‐operated mice, measured 28 days post surgery. One‐way ANOVA with Tukey multiple comparisons test (*n* = 7). (G) Immunofluorescence staining of CD31 (red), cTnI (green), and DAPI (blue) in cross‐sections of mouse hearts in indicated groups on day 28 after MI. Scale bar, 20 µm. (H) Quantitative analysis of microvessel density in border areas of hearts in Panel G. One‐way ANOVA with Tukey multiple comparisons test (*n* = 7).

To determine the therapeutic potential of peptide 7 in post‐infarction cardiac recovery, Rhodamine B–conjugated peptide 7 was administered intraperitoneally for the first 3 days post‐MI. Subsequent immunofluorescence microscopy confirmed its presence in cardiac macrophages (Figure S9A). To visualize its systemic delivery and tissue distribution, we performed ex vivo fluorescence imaging using Cy5.5‐conjugated peptides. The labeled peptide demonstrated broad biodistribution across multiple organs, including the heart, and significant fluorescence signal remained detectable at 12 h post administration (Figure S9B). This tissue distribution and time‐course profile confirm on‐target exposure in the heart and support sustained bioavailability within the therapeutic window. In therapeutic efficacy studies, WT mice were randomly assigned to three groups receiving either peptide 7, peptide ctrl, or vehicle. Injections were administered thrice weekly following MI. Serial echocardiography assessed cardiac function parameters (LVEDV, LVESV, EF, and FS) at preoperative baseline (day 0) and post‐MI intervals (days 3, 7, 14, and 28). Notably, mice treated with peptide 7 showed significant preservation of cardiac function compared to controls, as demonstrated by lower LVEDV and LVESV, and higher EF and FS (Figure 8A,B and Figure S9C). Furthermore, on 28 days post MI, peptide 7‐treated mice exhibited significantly reduced fibrotic areas compared to vehicle‐treated mice (Figure 8C,D). Additionally, the HW:BW and LW:BW were lower in peptide 7–treated mice (Figure 8E,F). In contrast, mice injected with peptide 7 exhibited significantly higher microvessel density in the infarcted border zone 28 days post‐MI compared to controls (Figure 8G,H).

Safety assessment revealed comparable tissue architecture in hepatic, renal, and intestinal specimens across experimental cohorts, with no evidence of vacuolization, hemosiderin deposition, or coagulative necrosis (Figure S9D). Serum biochemistry profiling revealed no significant intergroup variations in critical organ function markers: creatinine (Cr), blood urea nitrogen (BUN), alanine aminotransferase (ALT), and aspartate aminotransferase (AST) (Figure S9E). These collective findings demonstrate that the administration of peptide 7 at therapeutic concentrations maintains hepatorenal homeostasis and exhibits a favorable biosafety profile in murine models, with no detectable off‐target organ toxicity within the therapeutic window.

The above results suggest that peptide 7, which inhibits the HSC70–USP9X interaction in macrophages, effectively promotes cardiac repair and functional recovery post‐MI, demonstrating efficacy and safety in murine models.

## 3 Discussion

Our study identifies the deubiquitinase USP9X as a CMA‐calibrated molecular switch that governs the transition from pro‐inflammatory to reparative identity in macrophages following MI. USP9X expression in cardiac macrophages shows an initial decline followed by partial recovery after MI, aligning with the biphasic nature of post‐MI inflammation [60, 61]. We identified signaling by DAMPs released from necrotic cardiomyocytes as the upstream trigger for this initial depletion. Specifically, the prototypical DAMP HMGB1, acting through pathways such as TLR4 activation, triggered a time‐dependent decrease in USP9X protein levels in macrophages. This effect was recapitulated in vitro by LPS, a known TLR4 ligand. Together, these findings suggest that DAMP‐mediated signaling, which mimics the early post‐MI inflammatory milieu, is a key initiating event that drives USP9X loss. Genetic and pharmacological inhibition of USP9X in macrophages amplified pro‐inflammatory gene expression and exacerbated cardiac dysfunction. Conversely, stabilization of USP9X through inhibition of its CMA‐dependent degradation mitigates inflammation and improves functional recovery. These discoveries expand the understanding of DUB‐mediated cardiac pathophysiology and highlight USP9X as a promising therapeutic target for post‐infarction heart failure.

To elucidate the downstream molecular mechanisms by which USP9X depletion exacerbates inflammation and to address the persistent knowledge gaps regarding USP9X substrates and cascades in post‐MI cardiac macrophages, we performed proteomic and ubiquitinomic profiling. Our analysis revealed that USP9X ablation significantly upregulated pro‐inflammatory mediators while downregulating anti‐inflammatory proteins, consistent with both our phenotypic observations in cellular and animal models and prior reports [23, 62]. Through cross‐omics profiling and biochemical validation, we establish TRAFD1 as a deubiquitylation substrate of USP9X. TRAFD1, an established negative regulator of Toll‐like‐receptor‐mediated NF‐κB signaling, exhibited markedly accelerated proteasomal turnover in USP9X‐deficient macrophages, providing a mechanistic link between USP9X loss and inflammatory dysregulation.

While our study identifies TRAFD1 stabilization as a key mechanism by which USP9X restrains inflammation in cardiac macrophages, the molecular consequences of USP9X depletion are known to be multifaceted. Prior work indicates USP9X ablation exacerbates pro‐inflammatory responses through disrupting regulators like transformation/transcription domain–associated protein (TRRAP) in glioblastoma models [62]. However, USP9X is dispensable for PKCθ‐driven NF‐κB activation in T cells, highlighting the context‐dependent functions of this deubiquitinase [63]. Therefore, our work defines the USP9X–TRAFD1 axis as a critical and previously missing piece in understanding macrophage‐driven post‐MI remodeling.

The context‐dependency functions of USP9X are further illustrated by recent work. Li et al. demonstrated that USP9X promotes cardiac fibrosis via ARK5 activation in fibroblasts during the later reparative phase, functioning as a “fibrotic engine” [64]. In contrast, we found that in macrophages during the early inflammatory phase, USP9X stabilizes TRAFD1 to restrain inflammation, acting as an “inflammatory brake.” Rather than being contradictory, these observations reflect a fundamental property of USP9X as a substrate‐ and cell type–dependent deubiquitinase. This principle is also well established in oncology, where USP9X can function either as an oncogene or a tumor suppressor depending on cellular context [65]. Even within a single signaling cascade, such as the Hippo pathway, USP9X exerts opposing effects by stabilizing both tumor‐suppressive LATS2 and oncogenic YAP [66, 67]. Accordingly, the biological outcome of USP9X is determined by its selective substrates in a given context: TRAFD1 as an anti‐inflammatory target in macrophages versus ARK5 as a profibrotic target in fibroblasts. Together, these studies delineate a spatiotemporal functional landscape for USP9X in post‐MI remodeling, where its role is precisely defined by cell type and disease stage.

Having established that USP9X loss exacerbates cardiac macrophage inflammation via TRAFD1 destabilization, and that DAMP signaling initiates its depletion, we next sought to elucidate the precise degradative pathway. We found that USP9X degradation is mediated by CMA, which is initiated by sirtuin‐regulated acetylation at K2414. This modification exposes latent KFERQ motifs, enabling HSC70 recognition and subsequent lysosomal degradation during the early inflammatory phase. This model aligns with the established role of sirtuins in inhibiting inflammation, as their downregulation under pro‐inflammatory conditions would promote USP9X acetylation and degradation, thereby amplifying the inflammatory response [68, 69, 70, 71].

Building upon the critical role of the CMA–USP9X axis in post‐MI inflammation and the recognition that CMA itself is an important regulator of cardiovascular homeostasis [72, 73], therapeutic modulation of CMA is necessary. Since global CMA inhibition can be detrimental [74], we developed a strategy to selectively block the degradation of USP9X using a cell‐penetrating peptide that mimics its acetylated KFERQ motif. This peptide stabilized USP9X, promoted an anti‐inflammatory macrophage phenotype, and improved cardiac outcomes in mice, demonstrating the promise of targeted proteostasis modulation. This approach distinguishes USP9X from other DUBs implicated in cardiac remodeling. While many DUBs, such as MYSM1 and USP25 [12, 13, 75, 76, 77, 78, 79], primarily affect cardiomyocyte survival and intramyocardial inflammatory cascades, or others like USP10 and OTUB1 modulate fibrosis [14, 15, 16]. USP9X stands apart by uniquely orchestrating macrophage polarization through a CMA‐dependent mechanism. This pathway is seldom explored in cardiovascular DUB biology, highlighting a novel therapeutic angle.

While this study provides substantial evidence, several limitations warrant consideration. First, the roles of USP9X in non‐macrophage cell types within the heart remain unexplored. Understanding these broader effects is necessary for a comprehensive evaluation of USP9X's impact on cardiac repair and remodeling. Second, it is important to note that the pharmacological inhibitor WP1130 can inhibit multiple deubiquitinases. Therefore, the key conclusions of this study rest primarily on genetic evidence from macrophage‐specific *Usp9x* knockout models, which specifically establish the nonredundant role of USP9X in macrophages. Third, our analysis of macrophage subsets, while informative, does not definitively pinpoint which subpopulation is most functionally impacted at a single‐cell level. We hypothesize that the newly recruited monocytes and their derivatives are likely the key targets, given their dominance in the early infarct and association with a pro‐inflammatory state. Higher‐resolution mapping in a cell‐type‐specific knockout model would be required to precisely dissect subset‐specific functions and test this hypothesis. Finally, the long‐term safety, bioavailability and potential off‐target effects of peptide 7 remain to be rigorously evaluated before any clinical translation.

Therefore, future studies should focus on several key areas. To elucidate the cell‐specific roles of USP9X and its interactomes across cardiac cell subtypes, employing single‐cell proteomics will be critical. Concurrently, exploring combinatorial strategies targeting complementary pathways, such as other DUBs involved in fibrosis, immune modulation or metabolic reprogramming, may achieve synergistic therapeutic effects. Additionally, integrating cardiac‐targeting peptides with nanoparticle delivery systems could address issues of drug distribution and bioavailability. This combined approach holds promise for more precise and effective therapies for post‐MI cardiac repair.

In conclusion, this study establishes USP9X as a central regulator orchestrating the macrophage phenotypic switch during post‐MI cardiac repair. We mechanistically link the inflammatory‐reparative balance to USP9X abundance, which is controlled by its sirtuin‐mediated acetylation and subsequent CMA‐dependent degradation. The protective peptide we developed to block this degradation highlights the therapeutic potential of precisely targeting DUB dynamics, offering a novel strategy to improve outcomes in ischemic heart disease.

## 4 Experimental Section

The complete and detailed materials and methods are provided in the Supporting Information.

### 4.1 Animal Studies

All animal experiments were conducted in accordance with the Guide for the Care and Use of Laboratory Animals and approved by the Animal Ethics Committee of the Second Affiliated Hospital, Zhejiang University School of Medicine (Ethics Approval Number: (2025niandu)di(010)hao)). All mice were bred on a C57BL/6 background and maintained under a 12:12 h light/dark cycle (lights on at 7:00 and off at 19:00) before and during experiments. *Usp9x^fl/fl^* mice were kindly provided by Dr. Stephen A. Wood (Griffith University, Brisbane, Australia). We generated myeloid cell‐specific *Usp9x* knockout mice (Mac‐*Usp9x* KO) by crossing *Usp9x^fl/fl^* mice with transgenic mice expressing Cre recombinase under the control of a LysM promoter (Jackson Laboratories; stock #004781). MI model was performed on 8‐week‐old male mice by ligation of the left anterior descending coronary artery [80]. Mice were sacrificed by terminal anesthesia on day 3, 7 or 28 post MI, and hearts were collected for further analysis. WT mice (male, 8 weeks old) were pretreated with WP1130 (10 mg/kg, 100 µL, twice weekly, intraperitoneal injection) for 1 week, then subjected to MI surgery and treated with WP1130 for another 28 days. In some experiments, WT mice (male, 8 weeks old) were treated with peptides (20 mg/kg, 100 µL, three times a week, intraperitoneal injection) for 28 days following MI surgery. To minimize potential bias, all animal studies were conducted in a blinded manner. The investigators performing the MI surgery, subsequent drug treatments, echocardiographic assessments, and histological analyses were unaware of the genotype (e.g., *Usp9x^fl/fl^* vs. Mac‐*Usp9x* KO) and treatment group (e.g., peptide ctrl vs. peptide 7) allocation until after all data collection and primary analyses were completed.

### 4.2 Echocardiography

Echocardiographic assessments were performed on mice using a Vevo 2100 Imaging System (VisualSonics, Ontario, Canada) equipped with an MS‐400 imaging transducer. Mice were anesthetized with 1% isoflurane in 1.0 L/min O_2_. Two‐dimensional and M‐mode images of the left ventricle were captured at the level of the papillary muscles. LV dimensions, including diastolic and systolic wall thickness, as well as the LV internal diameter at end‐diastole and end‐systole, were measured from two‐dimensional short‐axis views using M‐mode tracings. Additionally, B‐mode tracings of the LV endocardial border in a parasternal long axis were performed to directly evaluate LVEDV and LVESV. These calculations were performed using the primary measurements and the accompanying software of the imaging system.

### 4.3 Cell Culture and Stably Transfected Cell Line Generation

Bone marrow cells were isolated as described previously [81] and then incubated for 7 days with murine M‐CSF (50 ng/mL) to induce BMDM differentiation. Peritoneal macrophages were harvested from mice 3 days after intraperitoneal injection of 3% thioglycolate as described previously [82]. Neonatal mouse cardiomyocytes or fibroblasts were isolated using Neonatal Heart Dissociation Kit (130‐098‐373, Miltenyi Biotec Inc.). HEK293 and RAW264.7 cells were obtained from the American Type Culture Collection (USA) and cultured according to the supplier's instructions. Cell lines were authenticated by morphology and growth characteristics and were confirmed to be mycoplasma‐free. SR‐A1‐WT/K27R‐EGFP stably transfected RAW264.7 cells were generated as described previously [23].

### 4.4 Immunoprecipitation

Cells were lysed in buffer containing protease inhibitors for 30 min at 4°C, then centrifuged at 10 000 × *g* for 15 min at 4°C. Specific antibodies and antibody‐conjugated magnetic beads (Bimake, USA) were added to the lysates and incubated for 12 h at 4°C with rotation. Subsequently, the antibody–lysate mixture was incubated with either protein A/G agarose beads (30 µL) for 2 h at room temperature. Immunoprecipitated proteins were eluted by boiling the beads in loading buffer for 5 min, then analyzed via WB.

### 4.5 Plasmids

The plasmids carrying HA‐Ub, HA‐Ub (K11), HA‐Ub (K48), the pLenti‐Tight‐Puro vector carrring FLAG‐tagged USP9X and pLenti‐Neo vector carrying rtTA were kindly provided by Dr. Lei Shi (Tianjin Medical University, Tianjin, China). Plasmids carrying FLAG‐tagged N‐terminal, middle (M), C1, or C2 motifs of USP9X were purchased from Shanghai Genechem Co. Ltd. (China). Plasmid carrying MYC‐tagged HSC70/TRAFD1 were purchased from Tsingke Biotechnology Co. Ltd. (China).

### 4.6 Peptide Synthesis and Delivery

The peptides were synthesized by Synpeptide Co. Ltd, China, via Fmoc solid‐phase peptide synthesis. The synthesis was performed on Wang resin, with amino acids coupled sequentially from the C‐terminus to the N‐terminus. Each coupling step was followed by deprotection using 20% piperidine in N,N ‐ dimethylformamide (DMF), and the progress was monitored by the ninhydrin test. After the final amino acid was coupled and deprotected, the peptides were cleaved from the resin using a trifluoroacetic acid (TFA)‐based cleavage cocktail and precipitated with cold diethyl ether. The peptides were then purified using high‐performance liquid chromatography (HPLC). The peptides were eluted with a linear gradient of water and acetonitrile (both containing 0.1% TFA) at a flow rate of 10 mL/min. Finally, the purified peptides were analyzed by mass spectrometry to confirm their molecular weights and purity. Inhibitory peptides targeting the interaction between USP9X and HSC70 were designed based on the KFERQ‐like motif in USP9X and listed in Table S1. To facilitate cellular uptake, an 11‐amino acid cell‐penetrating peptide (YGRKKRRQRRR) derived from the transduction domain of the TAT protein was chemically conjugated to the N‐terminus of the inhibitory peptides. For experimental tracking and validation, Rhodamine B–labeled inhibitory peptides were synthesized, with Rhodamine B conjugated to the N‐terminus and the cell‐penetrating peptide attached to the C‐terminus. These labeled peptides were used to assess peptide uptake and localization in mouse cardiac macrophages or cultured macrophages. In vitro, macrophages were treated with peptides at 20 µm. In vivo, the indicated peptides (20 mg/kg in saline) were administered to mice via intraperitoneal injection three times per week. To assess tissue distribution and time course, we synthesized peptides labeled with Cy5.5 at the N‐terminus and fused to a cell‐penetrating peptide at the C‐terminus. The peptide was administered via intraperitoneal injection (20 mg/kg in 100 µL) and its biodistribution was assessed by ex vivo fluorescence imaging.

### 4.7 Statistical Analysis

The number of replicates (*n*) for each experiment is indicated in the figure legends. Data preprocessing included normality assessment using the Shapiro–Wilk test (especially for sample sizes *n* < 10). For normally distributed data, comparisons between two groups were performed using unpaired or paired two‐tailed Student's *t*‐tests, while comparisons among multiple groups were analyzed using one‐way or two‐way ANOVA followed by Bonferroni posthoc tests or Tukey multiple comparisons test. For non‐normally distributed data, the Mann–Whitney *U*‐test was used. Specific tests for each experiment are detailed in the corresponding figure legend. Data are presented as mean ± standard error (SEM). Individual data points, representing technical replicates or biological individuals are shown where applicable. *p*‐values are shown in the figures, and *p* < 0.05 was considered statistically significant. All Statistical analyses were performed using GraphPad Prism (version 8.0).

## Funding

This research was supported by the National Natural Science Foundation of China (Grants 82300279, 82500972, 82200406, 82300497, 82130014, 82321001, 82430013, and 82200345), Fundamental Research Funds for the Central Universities (Grant 226‐2024‐00132), Shandong Provincial Natural Science Foundation (Grant ZR2025QC1668), Qingdao Natural Science Foundation (Grant 25‐1‐1‐144‐zyyd‐jch), the National Science Foundation of Zhejiang Province (Grant LQN25H020006), and the Natural Science Foundation of Tianjin, China (Grant 24ZXZSSS00430).

## Conflicts of Interest

The authors declare no conflict of interest.

## Supporting information




**Supporting File**: advs74037‐sup‐0001‐SuppMat.docx.

## Data Availability

The data that support the findings of this study are available from the corresponding author upon reasonable request.

## References

[1] S. S. Virani , A. Alonso , E. J. Benjamin , et al., “Heart Disease and Stroke Statistics‐2020 Update: A Report From the American Heart Association,” Circulation 141 (2020): e139–e596, 10.1161/cir.0000000000000757. 31992061

[2] N. Khalid , M. Abdullah , A. Afzal Muhammad , et al., “A Systematic Analysis of Global Disease Burden of Ischemic Heart Disease From 1990‐2019,” Journal of the American College of Cardiology 83 (2024): 1338–1338, 10.1016/S0735-1097(24)03328-X.38569764

[3] J. Feng , Y. Zhang , and J. Zhang , “Epidemiology and Burden of Heart Failure in Asia,” JACC: Asia 4 (2024): 249–264, 10.1016/j.jacasi.2024.01.013. 38660101 PMC11035951

[4] S. S. Dani , A. N. Lone , Z. Javed , et al., “Trends in Premature Mortality from Acute Myocardial Infarction in the United States, 1999 to 2019,” Journal of the American Heart Association 11 (2022): 021682, 10.1161/jaha.121.021682. PMC9075205 34935456

[5] G. M. Fröhlich , P. Meier , S. K. White , D. M. Yellon , and D. J. Hausenloy , “Myocardial Reperfusion Injury: Looking Beyond Primary PCI,” European Heart Journal 34 (2013): 1714–1722, 10.1093/eurheartj/eht090. 23536610

[6] J. T. Thackeray , H. C. Hupe , Y. Wang , et al., “Myocardial Inflammation Predicts Remodeling and Neuroinflammation after Myocardial Infarction,” Journal of the American College of Cardiology 71 (2018): 263–275, 10.1016/j.jacc.2017.11.024. 29348018

[7] P. C. Westman , M. J. Lipinski , D. Luger , et al., “Inflammation as a Driver of Adverse Left Ventricular Remodeling After Acute Myocardial Infarction,” Journal of the American College of Cardiology 67 (2016): 2050–2060, 10.1016/j.jacc.2016.01.073. 27126533

[8] J. Yap , J. Irei , J. Lozano‐Gerona , S. Vanapruks , T. Bishop , and W. A. Boisvert , “Macrophages in Cardiac Remodelling After Myocardial Infarction,” Nature Reviews Cardiology 20 (2023): 373–385, 10.1038/s41569-022-00823-5. 36627513

[9] S. C. Shen , J. Xu , C. Cheng , et al., “Macrophages Promote the Transition From Myocardial Ischemia Reperfusion Injury to Cardiac Fibrosis in Mice Through GMCSF/CCL2/CCR2 and Phenotype Switching,” Acta Pharmacologica Sinica 45 (2024): 959–974, 10.1038/s41401-023-01222-3. 38225394 PMC11053127

[10] S. Frantz , M. J. Hundertmark , J. Schulz‐Menger , F. M. Bengel , and J. Bauersachs , “Left Ventricular Remodelling Post‐Myocardial Infarction: Pathophysiology, Imaging, and Novel Therapies,” European Heart Journal 43 (2022): 2549–2561, 10.1093/eurheartj/ehac223. 35511857 PMC9336586

[11] J. Herrmann , W. D. Edwards , D. R. Holmes Jr. , et al., “Increased Ubiquitin Immunoreactivity in Unstable Atherosclerotic Plaques Associated With Acute Coronary Syndromes,” Journal of the American College of Cardiology 40 (2002): 1919–1927, 10.1016/s0735-1097(02)02564-0. 12475450

[12] S. Song , Y. Wang , H. Y. Wang , and L. L. Guo , “Role of Sevoflurane in Myocardial Ischemia‐Reperfusion Injury via the Ubiquitin‐Specific Protease 22/Lysine‐Specific Demethylase 3A Axis,” Bioengineered 13 (2022): 13366–13383, 10.1080/21655979.2022.2062535. 36700466 PMC9275884

[13] Y. Zhang , J. Hailati , X. Ma , H. Midilibieke , and Z. Liu , “Ubiquitin‐Specific Protease 11 Aggravates Ischemia‐Reperfusion‐Induced Cardiomyocyte Pyroptosis and Injury by Promoting TRAF3 Deubiquitination,” Balkan Medical Journal 40 (2023): 205–214, 10.4274/balkanmedj.galenos.2023.2022-12-15. 37000116 PMC10175892

[14] S. Xie , Y. Xing , W. Shi , et al., “Cardiac Fibroblast Heat Shock Protein 47 Aggravates Cardiac Fibrosis Post Myocardial Ischemia–Reperfusion Injury by Encouraging Ubiquitin Specific Peptidase 10 Dependent Smad4 Deubiquitination,” Acta Pharmaceutica Sinica B 12 (2022): 4138–4153, 10.1016/j.apsb.2022.07.022. 36386478 PMC9643299

[15] S. Yuan , Z. Wang , S. Yao , et al., “Knocking Out USP7 Attenuates Cardiac Fibrosis and Endothelial‐to‐Mesenchymal Transition by Destabilizing SMAD3 in Mice With Heart Failure With Preserved Ejection Fraction,” Theranostics 14 (2024): 5793–5808, 10.7150/thno.97767. 39346543 PMC11426239

[16] S. Li , M. Yang , Y. Zhao , et al., “Deletion of ASPP1 in Myofibroblasts Alleviates Myocardial Fibrosis by Reducing p53 Degradation,” Nature Communications 15 (2024): 8425, 10.1038/s41467-024-52739-y. PMC11439048 39341821

[17] M. Coggins and A. Rosenzweig , “The Fire Within: Cardiac Inflammatory Signaling in Health and Disease,” Circulation Research 110 (2012): 116–125, 10.1161/circresaha.111.243196. 22223209

[18] N. Shembade , A. Ma , and E. W. Harhaj , “Inhibition of NF‐κB Signaling by A20 Through Disruption of Ubiquitin Enzyme Complexes,” Science 327 (2010): 1135–1139, 10.1126/science.1182364. 20185725 PMC3025292

[19] M. Murtaza , L. A. Jolly , J. Gecz , and S. A. Wood , “La FAM Fatale: USP9X in Development and Disease,” Cellular and Molecular Life Sciences: CMLS 72 (2015): 2075–2089, 10.1007/s00018-015-1851-0. 25672900 PMC4427618

[20] A. Jaiswal , K. Murakami , A. Elia , et al., “Therapeutic Inhibition of USP9x‐Mediated Notch Signaling in Triple‐Negative Breast Cancer,” Proceedings of the National Academy of Sciences of the United States of America 118 (2021): 2101592118, 10.1073/pnas.2101592118. PMC8463885 34518219

[21] Y. Wang , C. Wang , F. Yang , et al., “USP9X‐Enriched MSC‐sEV Inhibits LSEC Angiogenesis in MASH Mice by Downregulating the IκBα/NF‐κB/Ang‐2 Pathway,” Pharmacological Research 209 (2024): 107471, 10.1016/j.phrs.2024.107471. 39427871

[22] Y. Lei , J. Hu , J. Zhao , et al., “Deubiquitinase USP9X Controls Wnt Signaling for CNS Vascular Formation and Barrier Maintenance,” Developmental Cell 60 (2025): 1618–1635, 10.1016/j.devcel.2025.01.009. 39909046

[23] B. Wang , X. Tang , L. Yao , et al., “Disruption of USP9X in Macrophages Promotes Foam Cell Formation and Atherosclerosis,” Journal of Clinical Investigation 132 (2022): 154217, 10.1172/jci154217. PMC9106359 35389885

[24] J. M. Tsai , R. P. Nowak , B. L. Ebert , and E. S. Fischer , “Targeted Protein Degradation: From Mechanisms to Clinic,” Nature Reviews Molecular Cell Biology 25 (2024): 740–757, 10.1038/s41580-024-00729-9. 38684868

[25] L. Zhao , J. Zhao , K. Zhong , A. Tong , and D. Jia , “Targeted Protein Degradation: Mechanisms, Strategies and Application,” Signal Transduction and Targeted Therapy 7 (2022): 113, 10.1038/s41392-022-00966-4. 35379777 PMC8977435

[26] S. Kaushik and A. M. Cuervo , “The Coming of Age of Chaperone‐Mediated Autophagy,” Nature Reviews Molecular Cell Biology 19 (2018): 365–381, 10.1038/s41580-018-0001-6. 29626215 PMC6399518

[27] A. M. Cuervo and E. Wong , “Chaperone‐Mediated Autophagy: Roles in Disease and Aging,” Cell Research 24 (2014): 92–104, 10.1038/cr.2013.153. 24281265 PMC3879702

[28] J. Madrigal‐Matute , J. de Bruijn , K. van Kuijk , et al., “Protective Role of Chaperone‐Mediated Autophagy Against Atherosclerosis,” Proceedings of the National Academy of Sciences of the United States of America 119 (2022): 2121133119, 10.1073/pnas.2121133119. PMC9168839 35363568

[29] J. Madrigal‐Matute , A. M. Cuervo , and J. C. Sluimer , “Chaperone‐Mediated Autophagy Protects Against Atherosclerosis,” Autophagy 18 (2022): 2505–2507, 10.1080/15548627.2022.2096397. 35787098 PMC9542634

[30] J. Shao , X. Lin , H. Wang , et al., “Targeted Degradation of Cell Surface Proteins via Chaperone Mediated Autophagy by Using Peptide Conjugated Antibodies,” Angewandte Chemie, International Edition in English 63 (2024): 202319232, 10.1002/anie.202319232. 38472118

[31] G. Zhong , X. Chang , W. Xie , and X. Zhou , “Targeted Protein Degradation: Advances in Drug Discovery and Clinical Practice,” Signal Transduction and Targeted Therapy 9 (2024): 308, 10.1038/s41392-024-02004-x. 39500878 PMC11539257

[32] C. Rocha‐Resende , F. Pani , and L. Adamo , “B Cells Modulate the Expression of MHC‐II on Cardiac CCR2−Macrophages,” Journal of Molecular and Cellular Cardiology 157 (2021): 98–103, 10.1016/j.yjmcc.2021.05.003. 33971183 PMC8319082

[33] D. Jia , S. Chen , P. Bai , et al., “Cardiac Resident Macrophage‐Derived Legumain Improves Cardiac Repair by Promoting Clearance and Degradation of Apoptotic Cardiomyocytes after Myocardial Infarction,” Circulation 145 (2022): 1542–1556, 10.1161/circulationaha.121.057549. 35430895

[34] I. Hilgendorf , S. Frantz , and N. G. Frangogiannis , “Repair of the Infarcted Heart: Cellular Effectors, Molecular Mechanisms and Therapeutic Opportunities,” Circulation Research 134 (2024): 1718–1751, 10.1161/circresaha.124.323658. 38843294 PMC11164543

[35] Y. Yang , J. Lv , S. Jiang , et al., “The Emerging Role of Toll‐Like Receptor 4 in Myocardial Inflammation,” Cell Death & Disease 7 (2016): 2234, 10.1038/cddis.2016.140. PMC4917669 27228349

[36] H. Yang , H. S. Hreggvidsdottir , K. Palmblad , et al., “A Critical Cysteine Is Required for HMGB1 Binding to Toll‐Like Receptor 4 and Activation of Macrophage Cytokine Release,” Proceedings of the National Academy of Sciences of the United States of America 107 (2010): 11942–11947, 10.1073/pnas.1003893107. 20547845 PMC2900689

[37] M. Andrassy , H. C. Volz , J. C. Igwe , et al., “High‐Mobility Group Box‐1 in Ischemia‐Reperfusion Injury of the Heart,” Circulation 117 (2008): 3216–3226, 10.1161/circulationaha.108.769331. 18574060

[38] M. Ma , W. Jiang , and R. Zhou , “DAMPs and DAMP‐Sensing Receptors in Inflammation and Diseases,” Immunity 57 (2024): 752–771, 10.1016/j.immuni.2024.03.002. 38599169

[39] C. Farah , L. Y. M. Michel , and J. L. Balligand , “Nitric Oxide Signalling in Cardiovascular Health and Disease,” Nature Reviews Cardiology 15 (2018): 292–316, 10.1038/nrcardio.2017.224. 29388567

[40] J. R. Kingery , T. Hamid , R. K. Lewis , et al., “Leukocyte iNOS Is Required for Inflammation and Pathological Remodeling in Ischemic Heart Failure,” Basic Research in Cardiology 112 (2017): 19, 10.1007/s00395-017-0609-2. 28238121 PMC5555149

[41] R. Mashima , K. Saeki , D. Aki , et al., “FLN29, a Novel Interferon‐ and LPS‐inducible Gene Acting as a Negative Regulator of Toll‐Like Receptor Signaling,” Journal of Biological Chemistry 280 (2005): 41289–41297, 10.1074/jbc.M508221200. 16221674

[42] T. Sanada , G. Takaesu , R. Mashima , R. Yoshida , T. Kobayashi , and A. Yoshimura , “FLN29 Deficiency Reveals Its Negative Regulatory Role in the Toll‐Like Receptor (TLR) and Retinoic Acid‐Inducible Gene I (RIG‐I)‐Like Helicase Signaling Pathway,” Journal of Biological Chemistry 283 (2008): 33858–33864, 10.1074/jbc.M806923200. 18849341 PMC2662213

[43] P. Hubel , C. Urban , V. Bergant , et al., “A Protein‐Interaction Network of Interferon‐Stimulated Genes Extends the Innate Immune System Landscape,” Nature Immunology 20 (2019): 493–502, 10.1038/s41590-019-0323-3. 30833792

[44] P. Paudel , Q. Zhang , C. Leung , et al., “Crystal Structure and Activity‐Based Labeling Reveal the Mechanisms for Linkage‐Specific Substrate Recognition by Deubiquitinase USP9X,” Proceedings of the National Academy of Sciences of the United States of America 116 (2019): 7288–7297, 10.1073/pnas.1815027116. 30914461 PMC6462090

[45] F. A. Mallette and S. Richard , “K48‐Linked Ubiquitination and Protein Degradation Regulate 53BP1 Recruitment at DNA Damage Sites,” Cell Research 22 (2012): 1221–1223, 10.1038/cr.2012.58. 22491476 PMC3411168

[46] M. L. Matsumoto , K. E. Wickliffe , K. C. Dong , et al., “K11‐Linked Polyubiquitination in Cell Cycle Control Revealed by a K11 Linkage‐Specific Antibody,” Molecular Cell 39 (2010): 477–484, 10.1016/j.molcel.2010.07.001. 20655260

[47] M. Chaurasia , S. Gupta , A. Das , B. S. Dwarakanath , A. Simonsen , and K. Sharma , “Radiation Induces EIF2AK3/PERK and ERN1/IRE1 Mediated Pro‐Survival Autophagy,” Autophagy 15 (2019): 1391–1406, 10.1080/15548627.2019.1582973. 30773986 PMC6613886

[48] L. A. Huber and D. Teis , “Lysosomal Signaling in Control of Degradation Pathways,” Current Opinion in Cell Biology 39 (2016): 8–14, 10.1016/j.ceb.2016.01.006. 26827287

[49] Q. Li , T. Yue , X. Du , et al., “HSC70 Mediated Autophagic Degradation of Oxidized PRL2 Is Responsible for Osteoclastogenesis and Inflammatory Bone Destruction,” Cell Death & Differentiation 30 (2023): 647–659, 10.1038/s41418-022-01068-y. 36182990 PMC9984420

[50] B. Li , H. Ming , S. Qin , et al., “HSPA8 Activates Wnt/β‐Catenin Signaling to Facilitate BRAF V600E Colorectal Cancer Progression by CMA‐Mediated CAV1 Degradation,” Advanced Science (Weinheim, Baden‐Wurttemberg, Germany) 11 (2024): 2306535, 10.1002/advs.202306535. 37973552 PMC10797426

[51] L. Wang , J. Cai , X. Zhao , et al., “Palmitoylation Prevents Sustained Inflammation by Limiting NLRP3 Inflammasome Activation Through Chaperone‐Mediated Autophagy,” Molecular Cell 83 (2023): 281–297, 10.1016/j.molcel.2022.12.002. 36586411

[52] Y. Zhang , Y. Y. Xu , C. B. Yao , et al., “Acetylation Targets HSD17B4 for Degradation via the CMA Pathway in Response to Estrone,” Autophagy 13 (2017): 538–553, 10.1080/15548627.2016.1268302. 28296597 PMC5361611

[53] P. Kirchner , M. Bourdenx , J. Madrigal‐Matute , et al., “Proteome‐Wide Analysis of Chaperone‐Mediated Autophagy Targeting Motifs,” PLoS Biology 17 (2019): 3000301, 10.1371/journal.pbio.3000301. PMC6561683 31150375

[54] J. Li , L. Lu , L. Liu , et al., “HDAC1/2/3 are Major Histone Desuccinylases Critical for Promoter Desuccinylation,” Cell Discovery 9 (2023): 85, 10.1038/s41421-023-00573-9. 37580347 PMC10425439

[55] W. Wang , R. L. Xu , P. He , and R. Chen , “MAR1 Suppresses Inflammatory Response in LPS‐Induced RAW 264.7 Macrophages and Human Primary Peripheral Blood Mononuclear Cells via the SIRT1/PGC‐1α/PPAR‐γ Pathway,” Journal of Inflammation (London, England) 18 (2021): 8, 10.1186/s12950-021-00271-x. 33557833 PMC7869219

[56] S. Han , Z. Li , P. Ji , et al., “MCPIP1 Alleviated Lipopolysaccharide‐Induced Liver Injury by Regulating SIRT1 via Modulation of Microrna‐9,” Journal of Cellular Physiology 234 (2019): 22450–22462, 10.1002/jcp.28809. 31099043

[57] S. H. Chan , C. H. Hung , J. Y. Shih , et al., “SIRT1 Inhibition Causes Oxidative Stress and Inflammation in Patients With Coronary Artery Disease,” Redox Biology 13 (2017): 301–309, 10.1016/j.redox.2017.05.027. 28601780 PMC5466584

[58] Y. Hirata , Y. Yamada , S. Taguchi , et al., “Conjugated Fatty Acids Drive Ferroptosis Through Chaperone‐Mediated Autophagic Degradation of GPX4 by Targeting Mitochondria,” Cell Death & Disease 15 (2024): 884, 10.1038/s41419-024-07237-w. 39643606 PMC11624192

[59] C. Park , Y. Suh , and A. M. Cuervo , “Regulated Degradation of Chk1 by Chaperone‐Mediated Autophagy in Response to DNA Damage,” Nature Communications 6 (2015): 6823, 10.1038/ncomms7823. PMC4400843 25880015

[60] S. D. Prabhu and N. G. Frangogiannis , “The Biological Basis for Cardiac Repair After Myocardial Infarction,” Circulation Research 119 (2016): 91–112, 10.1161/circresaha.116.303577. 27340270 PMC4922528

[61] A. J. Mouton , K. Y. DeLeon‐Pennell , O. J. Rivera Gonzalez , et al., “Mapping Macrophage Polarization Over the Myocardial Infarction Time Continuum,” Basic Research in Cardiology 113 (2018): 26, 10.1007/s00395-018-0686-x. 29868933 PMC5986831

[62] B. Mu , J. Jing , R. Li , and C. Li , “USP9X Deubiquitinates TRRAP to Promote Glioblastoma Cell Proliferation and Migration and M2 Macrophage Polarization,” Naunyn‐Schmiedeberg's Archives of Pharmacology 398 (2025): 855–865, 10.1007/s00210-024-03313-2. 39073416

[63] E. Naik , J. D. Webster , J. DeVoss , J. Liu , R. Suriben , and V. M. Dixit , “Regulation of Proximal T Cell Receptor Signaling and Tolerance Induction by Deubiquitinase Usp9X,” Journal of Experimental Medicine 211 (2014): 1947–1955, 10.1084/jem.20140860. 25200027 PMC4172213

[64] X. Li , S. Jiang , W. Yang , et al., “Ubiquitin Specific Protease 9X Regulates the Activation of ARK5 and Promotes Progression of Fibrotic Remodeling,” JACC: Basic to Translational Science 10 (2025): 101255, 10.1016/j.jacbts.2025.02.014. 40310323 PMC12434208

[65] H. Gao , Z. Chen , L. Zhao , C. Ji , and F. Xing , “Cellular Functions, Molecular Signalings and Therapeutic Applications: Translational Potential of Deubiquitylating Enzyme USP9X as a Drug Target in Cancer Treatment,” Biochimica et Biophysica Acta (BBA)—Reviews on Cancer 1879 (2024): 189099, 10.1016/j.bbcan.2024.189099. 38582329

[66] C. Zhu , X. Ji , H. Zhang , et al., “Deubiquitylase USP9X Suppresses Tumorigenesis by Stabilizing Large Tumor Suppressor Kinase 2 (LATS2) in the Hippo Pathway,” Journal of Biological Chemistry 293 (2018): 1178–1191, 10.1074/jbc.RA117.000392. 29183995 PMC5787797

[67] L. Li , T. Liu , Y. Li , et al., “The Deubiquitinase USP9X Promotes Tumor Cell Survival and Confers Chemoresistance Through YAP1 Stabilization,” Oncogene 37 (2018): 2422–2431, 10.1038/s41388-018-0134-2. 29449692 PMC5940338

[68] K. Nakamura , M. Zhang , S. Kageyama , et al., “Macrophage Heme Oxygenase‐1‐SIRT1‐p53 Axis Regulates Sterile Inflammation in Liver Ischemia‐Reperfusion Injury,” Journal of Hepatology 67 (2017): 1232–1242, 10.1016/j.jhep.2017.08.010. 28842295 PMC5884687

[69] J. Xie , X. Zhang , and L. Zhang , “Negative Regulation of Inflammation by SIRT1,” Pharmacological Research 67 (2013): 60–67, 10.1016/j.phrs.2012.10.010. 23098819

[70] K. L. Mendes , D. F. Lelis , and S. H. S. Santos , “Nuclear Sirtuins and Inflammatory Signaling Pathways,” Cytokine & Growth Factor Reviews 38 (2017): 98–105, 10.1016/j.cytogfr.2017.11.001. 29132743

[71] Y. Yang , Y. Liu , Y. Wang , et al., “Regulation of SIRT1 and Its Roles in Inflammation,” Frontiers in Immunology 13 (2022): 831168, 10.3389/fimmu.2022.831168. 35359990 PMC8962665

[72] Y. Tang , M. Feng , Y. Su , et al., “Jmjd4 Facilitates Pkm2 Degradation in Cardiomyocytes and Is Protective Against Dilated Cardiomyopathy,” Circulation 147 (2023): 1684–1704, 10.1161/circulationaha.123.064121. 37066795

[73] M. Liu , S. Li , M. Yin , et al., “Pinacidil Ameliorates Cardiac Microvascular Ischemia‐Reperfusion Injury by Inhibiting Chaperone‐Mediated Autophagy of Calreticulin,” Basic Research in Cardiology 119 (2024): 113–131, 10.1007/s00395-023-01028-8. 38168863 PMC10837255

[74] L. Qiao , J. Ma , Z. Zhang , et al., “Deficient Chaperone‐Mediated Autophagy Promotes Inflammation and Atherosclerosis,” Circulation Research 129 (2021): 1141–1157, 10.1161/circresaha.121.318908. 34704457 PMC8638823

[75] X. Shi , J. Xu , L. Liu , et al., “Deubiquitinase MYSM1 Drives Myocardial Ischemia/Reperfusion Injury by Stabilizing STAT1 in Cardiomyocytes,” Theranostics 15 (2025): 1606–1621, 10.7150/thno.100097. 39897566 PMC11780537

[76] B. Ye , Y. Chen , X. Chen , et al., “Deubiquitinase USP25 Alleviates Obesity‐Induced Cardiac Remodeling and Dysfunction by Downregulating TAK1 and Reducing TAK1‐Mediated Inflammation,” JACC: Basic to Translational Science 9 (2024): 1287–1304, 10.1016/j.jacbts.2024.06.001. 39619140 PMC11604529

[77] Z. Wu , W. Li , S. Wang , and Z. Zheng , “Role of Deubiquitinase USP47 in Cardiac Function Alleviation and Anti‐Inflammatory Immunity After Myocardial Infarction by Regulating NLRP3 Inflammasome‐Mediated Pyroptotic Signal Pathways,” International Immunopharmacology 136 (2024): 112346, 10.1016/j.intimp.2024.112346. 38850785

[78] L. Liu , J. Pang , D. Qin , et al., “Deubiquitinase OTUD5 as a Novel Protector Against 4‐HNE‐Triggered Ferroptosis in Myocardial Ischemia/Reperfusion Injury,” Advanced Science (Weinheim, Baden‐Wurttemberg, Germany) 10 (2023): 2301852, 10.1002/advs.202301852. 37552043 PMC10558642

[79] H. Liang , X. Su , Q. Wu , et al., “LncRNA 2810403D21Rik/Mirf Promotes Ischemic Myocardial Injury by Regulating Autophagy Through Targeting Mir26a,” Autophagy 16 (2020): 1077–1091, 10.1080/15548627.2019.1659610. 31512556 PMC7469676

[80] E. van Rooij , “Cardiac Repair After Myocardial Infarction,” New England Journal of Medicine 374 (2016): 85–87, 10.1056/NEJMcibr1512011. 26735998

[81] X. Zhang , R. Goncalves , and D. M. Mosser , “The Isolation and Characterization of Murine Macrophages,” Current Protocols in Immunology 83 (2008): 14.1.11–14.1.14, 10.1002/0471142735.im1401s83. PMC2834554 19016445

[82] K. J. Moore , L. P. Andersson , R. R. Ingalls , et al., “Divergent Response to LPS and Bacteria in CD14‐Deficient Murine Macrophages,” The Journal of Immunology 165 (2000): 4272–4280, 10.4049/jimmunol.165.8.4272. 11035061

