# Supplementary material for: Chaperone‐Mediated Autophagic Degradation of USP9X in Macrophages Exacerbates Postmyocardial Infarction Inflammation and Cardiac Dysfunction
Source: Adv Sci (Weinh). 2026 Jan 28;13(19):e18950. doi: 10.1002/advs.202518950 (PMC13045210; doi:10.1002/advs.202518950)
Supplement: Supplementary file 1 — Supporting File: advs74037‐sup‐0001‐SuppMat.docx. [file ADVS-13-e18950-s001.docx]

**Supporting Information**

Figures S1 to 9

Tables S1 to 3

References (1 to 2)

**Title**

**Chaperone-Mediated Autophagic Degradation of USP9X in Macrophages Exacerbates Post-Myocardial Infarction Inflammation and Cardiac Dysfunction**

*Biqing Wang,^1,4^ Xiangheng Cai,^2^ Mengqi Li,^5^ Xue Liu,^6^ Junhui Xue,^1,4^ Ye Liu,^1,4^ Ding Ai,^3*^ Xinyang Hu^1,4*^*

^1^Department of Cardiology, The Second Affiliated Hospital, Zhejiang University School of Medicine, Zhejiang University, Hangzhou 310009, China.

^2^The Affiliated Hospital of Qingdao University, Qingdao 266100, China.

^3^The Province and Ministry Co-sponsored Collaborative Innovation Center for Medical Epigenetics, State Key Laboratory of Experimental Hematology, National Clinical Research Center for Blood Diseases, Key Laboratory of Immune Microenvironment and Disease (Ministry of Education), Tianjin Institute of Cardiology, The Second Hospital of Tianjin Medical University, Tianjin Medical University, Tianjin 300070, China.

^4^State Key Laboratory of Transvascular Implantation Devices, Heart Regeneration and Repair Key Laboratory of Zhejiang province, Binjiang Institute of Zhejiang University, Transvascular Implant Instrument Research Institute, Hangzhou 310009, China.

^5^Tianjin Medical University General Hospital, Tianjin 300070, China.

^6^Zhejiang Chinese Medical University, Hangzhou, 310053, China

Biqing Wang and Xiangheng Cai contributed equally to this work.

*^*^*Correspondence: Xinyang Hu ([hxy0507@zju.edu.cn](mailto:hxy0507@zju.edu.cn)), Ding Ai ([edin2000cn@163.com](mailto:edin2000cn@163.com)).

**Supplementary Methods**

Reagents

siRNA for *Usp9x* (sc-100628) and control siRNA (sc-37007) were obtained from Santa Cruz Biotechnology (USA). SiRNAs targeting *Hspa8, Trafad1 and Lamp2a* were purchased from Tsingke Biotechnology Co., Ltd. (China). Lysosome insolation kit (ab234047) was purchased from Abcam (USA). Masson’s Trichrome Stain Kit (G1346), Doxycycline (D8740) and LPS (L8880) were from Beijing Solarbio Science & Technology Co (China). Cycloheximide (HY-12320), bafilomycin A1 (HY-100558), 3-methyladenine (HY-19312), trichostatin A (HY-15144), nicotinamide (HY-B0150), HMGB1 ([HY-P72797](https://www.medchemexpress.cn/recombinant-proteins/hmgb1-hmg-1-protein-human-his.html)) and chloroquine (HY-17589A) were from MedChemExpress (China). WP1130 (S2243) and MG132 (S2619) were from Selleckchem (USA). NH_4_Cl (12125-02-9), corn oil (8001-30-7) and tribromoethanol (75-80-9) were from Aladdin Scientific Corp (China). Murine M-CSF (315-02) was purchased from Peprotech (USA). Antibodies against USP9X (55054-1-AP), NOS2 (22226-1-AP), TRAFD1 (27741-1-AP) and NFKBIE (11273-1-AP) were from Proteintech (China). Antibodies against USP9X (sc-36353, for immunofluorescence), acetylated lysine (sc-32268) and β-actin (sc-8432) were from Santa Cruz Biotechnology (USA). Antibodies against HSC70 (ET1602-33), GPX4 (ET1706-45) and CHK1(ET1609-71) were from HUABIO (China). Antibodies against CD68 (ab955), LAMP1 (ab208943), LAMP2A (ab125068) and cTnI (ab47003) were from Abcam (UK). Antibodies against FLAG (F1804) were from Sigma-Aldrich (USA). Antibodies against rabbit IgG (7074), HA (3724) and MYC (9B11) were from Cell Signaling Technology (USA). Anti-CD31 (AF3628) was from R&D Systems (USA). Antibody against pan phospho-Serine/Threonine (AP1067) was from ABclonal (China).

Mass spectrometry

Peritoneal macrophage lysates were prepared from 1 × 10^7^ cells. For IP, the USP9X antibody was incubated with total lysates, followed by adding Protein A/G PLUS-Agarose beads (Thermo Fisher Scientific, MA, USA). After binding, the agarose beads were washed with cold phosphate-buffered saline (PBS) containing 0.1% NP-40. The immunoprecipitated complex was separated on a 4-12% Bis-tris gel (SurePAGE, China) and stained with Coomassie Blue using a kit (22840, Thermo Fisher Scientific). Gel bands were excised and subjected to LC-MS/MS sequencing and data analysis as previously described [1] (performed by PTM Biolabs, China).

Protein extraction was performed from BMDMs stimulated with LPS for 12 h using urea lysis buffer (8 M urea, 1% protease inhibitor cocktail) followed by sonication and centrifugation. Proteins were reduced with 5 mM DTT (56 °C, 30 mins), alkylated with 15 mM iodoacetamide (room temperature, 15 min in darkness), and digested with trypsin (1:50 enzyme-to-protein ratio) at 37 °C for 16 h. For ubiquitination analysis, peptides were enriched with anti-ubiquitin antibody-conjugated agarose beads (PTMScan®), washed with IAP buffer (50 mM MOPS, 10 mM KH₂PO₄, 50 mM NaCl), and eluted with 0.15% TFA. LC-MS/MS analysis was performed using two complementary platforms (Cosmos wisdom, China). For ubiquitinome profiling, peptides were separated on a nanoflow LC system (Easy-nLC 1200) equipped with a reversed-phase C18 column (20 cm × 75 μm, 1.9 μm particles) with a 34-minute gradient from 7% to 32% acetonitrile/0.1% formic acid, followed by a 3-minute ramp to 80% acetonitrile at a constant flow rate of 450 mL/min. Mass spectrometry was conducted on a Q Exactive HF-X instrument operating in data-independent acquisition mode with the following parameters: MS1 scans at 400-1200 m/z (60,000 resolution, AGC target 3×10⁶), followed by MS2 scans at 100-1800 m/z (15,000 resolution, 24 m/z isolation windows, NCE 27%).

For global proteome analysis, we employed an Astral mass spectrometer coupled to a Vanquish Neo nanoLC system. The instrument was configured with MS1 scans at 380-980 m/z (240,000 resolution at 200 m/z) and MS2 acquisition using 150 variable windows (4 m/z isolation, 25 eV HCD) with automatic gain control targets set to 500% for both MS levels.

All mass spectrometry data were processed using DIA-NN (v1.8) against the appropriate UniProt database with the following parameters: trypsin/P specificity allowing up to 2 missed cleavages, fixed carbamidomethylation (C), variable ubiquitinylation (K), and a false discovery rate threshold of <1% at both protein and peptide levels. Bioinformatics analysis included functional enrichment of GO terms using Fisher's exact test (significance threshold *P* < 0.05).

Immunofluorescence staining

Cell or tissue sections were fixed in 4% paraformaldehyde for 30 min and washed three times with PBS. Following fixation, slides were permeabilized in PBS containing 0.05% Triton X-100 for 15 mins. Nonspecific binding sites blocked with 3% bovine serum albumin in PBS for 30 mins at room temperature. Slides were then incubated with primary antibody solution at 4 °C overnight. After washing three times with PBS, cells were incubated with Alexa Fluor 488 (ab150077, ab150113) or DyLight 550 (ab96876, ab96892, ab96932)-conjugated secondary antibodies (Abcam, USA) for 2 h at room temperature. Finally, slides were coverslipped using antifade mounting medium with DAPI (ZSGB-Bio, China). Fluorescence was visualized using confocal laser scanning microscopy.

Histological analysis

Mouse hearts were excised, briefly washed and arrested in diastole using a buffer containing 4.7 mM KCl and 0.1% 2,3-butanedione monoximein in PBS. Heart, liver, intestine, and kidney tissues were then fixed in 4% paraformaldehyde (pH 7.4) overnight. Following fixation, tissues were dehydrated through a graded ethanol series and embedded in paraffin. Paraffin-embedded blocks were sectioned at 4 µm thickness. Heart sections were stained with Sirius Red (Sbjbio, BP-DL029) to determine infarct size, while sections from the liver, intestine, and kidney were stained with hematoxylin and eosin (H & E) for histological analysis. Infarct size in the heart was calculated using the following formula: Infarct size (%) = (length of coronal infarct perimeter, including epicardial and endocardial surfaces) : (total left ventricular coronal perimeter, including epicardial and endocardial surfaces) × 100.

Quantification of liver and kidney function markers

Blood samples from mice were collected into serum separator tubes without anticoagulants and allowed to clot at room temperature for 30-60 mins. Serum was separated by centrifugation at 1000 g for 10 mins at 4 °C. Serum levels of BUN, Cr, AST and ALT were measured using commercial kits (Nanjing Jiancheng Bioengineering Institude, China) according to the manufacturer's instructions.

WB analysis

Cells or tissues were homogenized in cold RIPA lysis buffer supplemented with Protease and Phosphatase Inhibitor Cocktail (78442, Thermo Scientific). Equivalent amounts of proteins were denatured, resolved by SDS-polyacrylamide gel electrophoresis (SDS-PAGE) and transferred to nitrocellulose membranes. The membranes were blocked with 5% skim milk and incubated with primary antibodies overnight at 4 °C. Membranes were incubated with horseradish peroxidase–conjugated secondary antibody (7074/7076, CST) and detected using enhanced chemiluminescence (KF8003, Affinity Biosciences).

siRNA and plasmid transfection

Cells were seeded into plates at 70–80% confluency. For RNA interference, cells were transfected with gene-specific siRNA or control siRNA (10 nM) using Lipofectamine RNAi MAX (Thermo Fisher Scientific, USA) following the manufacturer's instructions. For plasmid transfection, Lipofectamine 3000 (Thermo Fisher Scientific, USA) was used according to the manufacturer's protocol. Fresh complete medium was replaced 6 h post-transfection, and cells were cultured for an additional 48 hours before treatment with the indicated reagents.

Lentivirus-mediated gene expression

Lentiviral constructs encoding TRAFD1 and FLAG-tagged USP9X^WT/K2414R^ were purchased from Shanghai GeneChem Co., Ltd. BMDMs were infected with the lentiviruses following the manufacturer’s instructions. Protein expression was analyzed 72 h after infection.

Total RNA isolation and quantitative real-time polymerase chain reaction

Total RNA was extracted from cells using the TransZol Up Plus RNA Kit (ER101-01, Transgen). Extracted RNA was reverse-transcribed into cDNA using the HiScript II Q Select RT SuperMix for qPCR (R233-01, Vazyme), following the manufacturer's instructions. Quantitative PCR was performed using the ChamQ SYBR qPCR Master Mix (Q341-02, Vazyme). The expression levels of target genes were normalized to those of housekeeping genes. All primer sequences used in this study are listed in **Table S2**.

MTT assay

Cells were seeded in 96-well plates and incubated for 24 hours to allow adherence. Subsequently, the culture medium was replaced with serum-free medium containing the indicated peptides, and the cells were incubated for the specified durations. Following peptide exposure, MTT solution (M1020, Beijing Solarbio Science & Technology Co) was added to each well, and cells were incubated for 4 h to allow formazan crystal formation. After removal of the MTT-containing medium, Formazan-dissolving reagent was added to dissolve the crystals. Absorbance was measured at 490 nm using a microplate reader. Cell viability was calculated as a percentage relative to the control group.

Mouse cardiac macrophage isolation

Cardiac macrophages were isolated using a protocol adapted from a previously published study.[2] Briefly, heart ventricles were dissected and minced into small pieces. The minced tissue was transferred to a centrifuge tube containing digestion buffer with collagenase type II (17101015, Thermo Fisher Scientific) and DNase I (EN0521, Thermo Fisher Scientific). The mixture was incubated at 37 °C for two 15-minute digestion cycles with gentle shaking. Isolated cells were resuspended and labeled with mouse anti-F4/80 magnetic microbeads (130-110-443, Miltenyi Biotec Inc.). Macrophages were collected using MS columns (130-042-201, Miltenyi Biotec Inc.) on an OctoMACS™ Separator (130-042-109, Miltenyi Biotec Inc.) and then lysed for WB analysis.

Flow cytometric analysis

Single-cell suspensions were prepared as described above. Cells were harvested, resuspended in buffer containing 2% fetal bovine serum, and incubated with antibodies specific to target surface antigens for 30 min at 4 °C. After washing to remove unbound antibodies, cells were fixed and permeabilized using an intracellular fixation & permeabilization buffer set (88-8824-00, Thermo Fisher Scientific Inc.) for intracellular staining. Following fixation and permeabilization, cells were incubated with antibodies targeting the intracellular protein of interest and washed extensively. Appropriate controls, including unstained cells and isotype controls, were included. Flow cytometry was performed on an LSR Fortessa instrument (BD Biosciences), and data were analyzed using FlowJo software. A sequential gating strategy was applied to analyze immune cells within the single-cell suspension. First, cells were plotted on a forward scatter area (FSC-A) vs side scatter area (SSC-A) plot to gate the live cell population and exclude debris. From this population, single cells were then gated based on forward scatter height (FSC-H) vs FSC-A to exclude doublets. Specific immune cell subsets were subsequently defined using fluorescently conjugated antibodies (see **Table S3** for antibody details).

**Supplementary Figures and Tables**

**
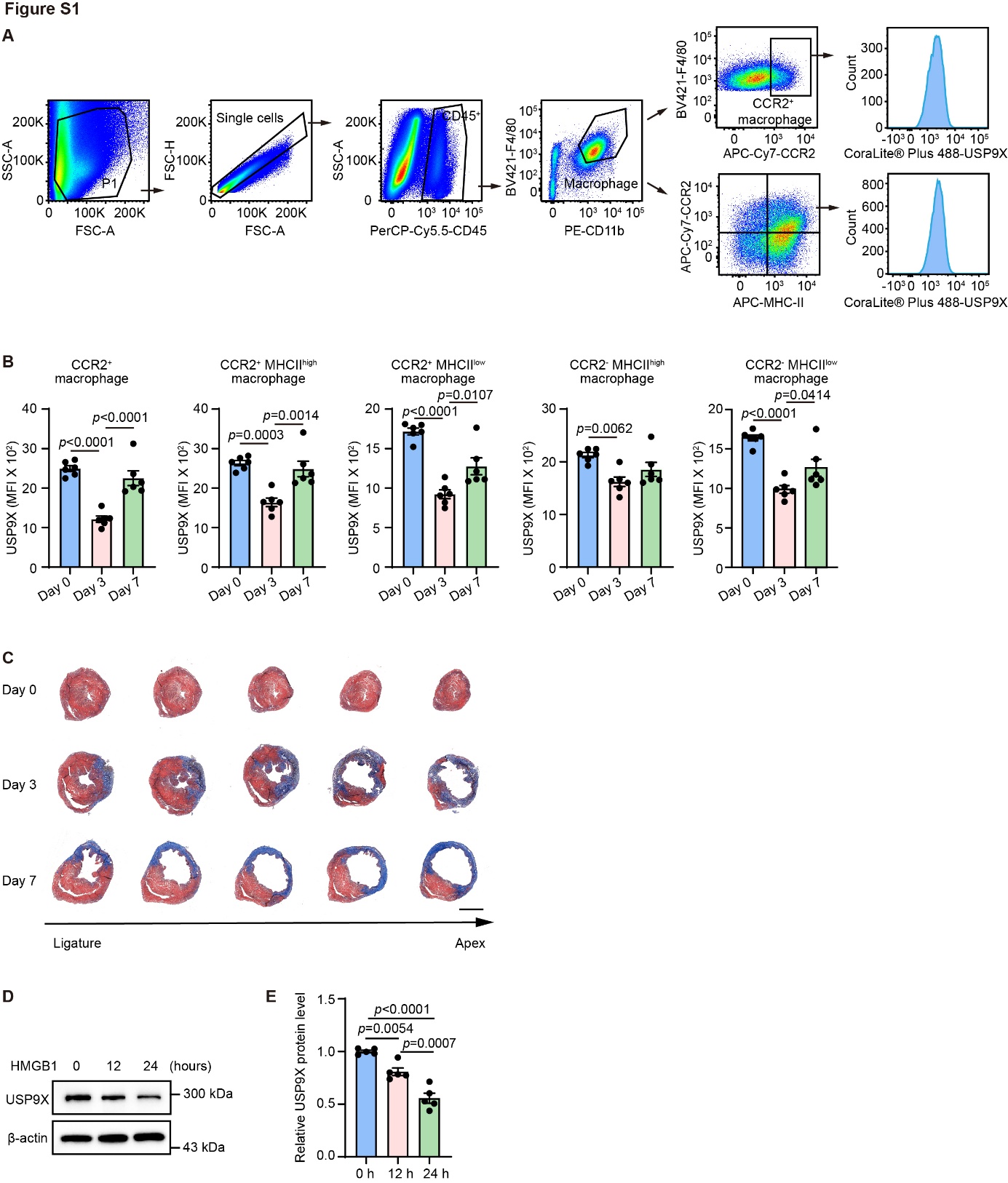
**

**Figure S1:**

(**A**) Representative flow-cytometry plots show the gating strategy and intracellular USP9X staining in cardiac macrophages from WT mice after MI. (**B**) Quantification of USP9X mean fluorescence intensity (MFI) in cardiac macrophages from WT mice at the indicated time points post-MI (n = 5)*.* (**C**) Representative Masson’s trichrome-stained heart cryosections from WT mice collected at 0, 3 and 7 day post-MI. Blue indicates fibrotic area; red indicates viable myocardium. Scale bar, 1 mm. (**D**) Western blot analysis of USP9X expression in BMDMs at indicated time points following stimulation with HMGB1 (1 µg/mL). (**E**) Quantification of USP9X levels shown in **D**. One-way ANOVA with Tukey’s multiple comparisons test (n = 5).

**
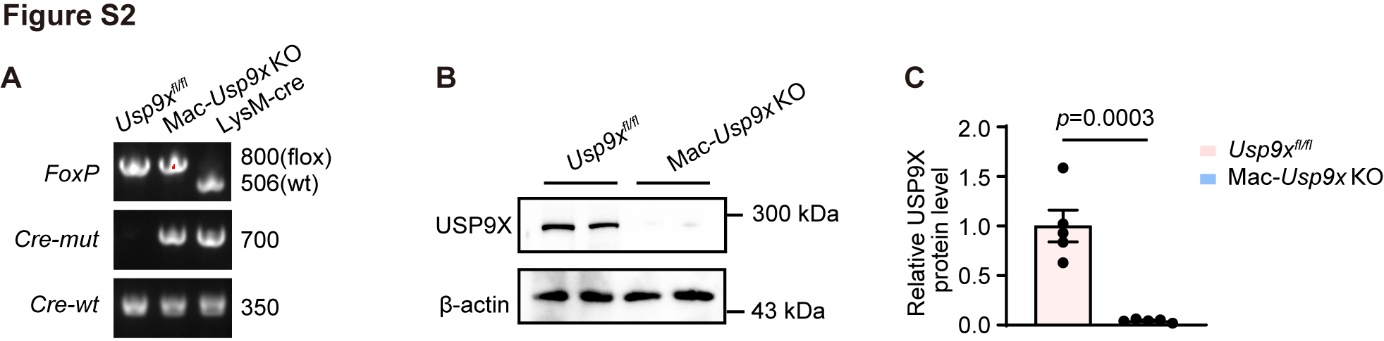
**

**Figure S2: Validation of macrophage-specific *Usp9x* knockout**

(**A**) Genotyping of *Usp9x^fl/fl^* mice, Mac-*Usp9x* KO mice, and LysM-Cre mice. (**B**) Western blot analysis of USP9X expression in BMDMs isolated from *Usp9x^fl/fl^* and Mac-*Usp9x* KO mice. (**C**) Quantification of USP9X protein levels shown in **B**. Student’s *t* test (n = 5).

**
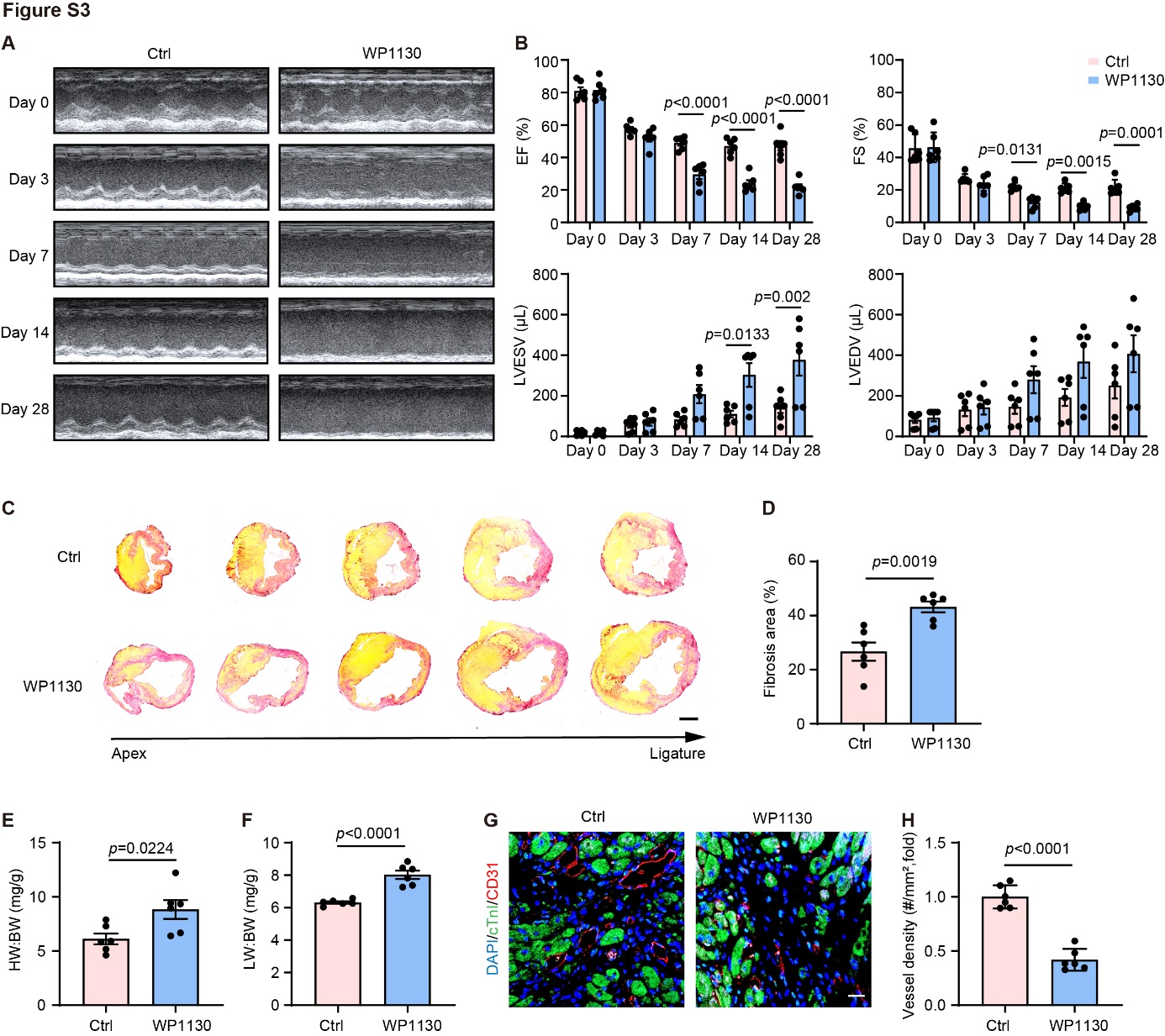
**

**Figure S3: Pharmacological inhibition of USP9X exacerbates cardiac dysfunction and impairs post-MI repair in mice**

WT mice were administered vehicle or WP1130 (10 mg/kg, 100 μL, twice weekly, via intraperitoneal injection) starting 1 week prior to the surgery and continuing for 28 days post-MI. (**A**) Representative M-mode echocardiograms from the indicated groups on days 0, 3, 7, 14 and 28 after MI from mice in indicated groups. (**B**) Echocardiographic measurements of EF, FS, LVEDV and LVESV in indicated groups. Two-way ANOVA with Tukey's multiple comparisons test (n = 6). (**C**) Representative Sirius red-stained cross-sections of hearts (cut at 200-μm intervals) from the indicated groups at day 28 post-MI. Scale bar, 1mm. (**D**) Quantification of infarct size percentage from **C**. Student’s *t* test (n = 6). (**E**) HW:BW in MI-operated mice, measured at 28 days post-surgery. Student’s *t* test (n = 6). (**F**) LW:BW in MI-operated mice, measured at 28 days post-surgery. Student’s *t* test (n = 6). (**G**) Representative immunofluorescence staining of CD31 (red), cTnI (green) and DAPI (blue) in heart cross-sections (border zone) from the indicated groups at day 28 post-MI. Scale bar, 20 μm. (**H**) Quantitative analysis of microvessel density in the border zone from **G**. Student’s *t* test (n = 6).


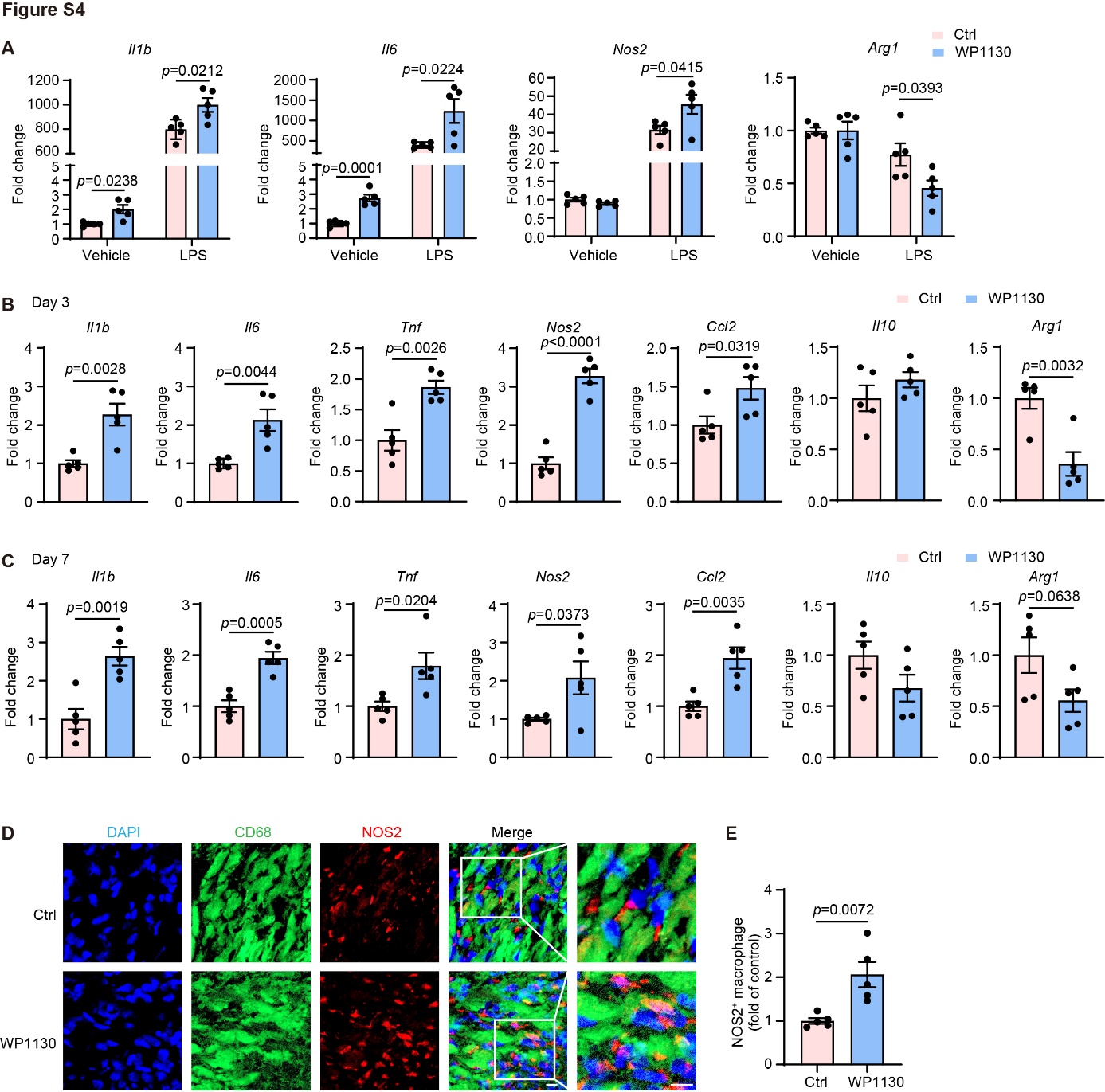


**Figure S4: Inhibition of USP9X in macrophages promotes their shift toward a pro-inflammatory phenotype**

(**A**) Quantitative PCR analysis of indicated gene expressions in BMDMs isolated from WT mice which were pre-treated with either vehicle or WP1130 (5 μM) and subsequently exposed to either LPS (100 ng/mL) or PBS for 24 h. Target gene expression was normalized to *Actb* mRNA levels. Two-way ANOVA with Tukey's multiple comparisons test (n = 5). (**B**-**C**) Quantitative PCR analysis of indicated gene expressions in heart tissues from vehicle or WP1130 treated mice at day 3 (**B**) and day 7 (**C**) post-MI. Target gene expression was normalized to *Actb* mRNA levels. Student’s *t* test (n = 5). (**D**) Representative immunofluorescence staining of NOS2 (red), CD68 (green) and DAPI (blue) in heart cryosections (border zone) from the indicated groups at day 3 post-MI. Scale bar, 20 μm. (**E**) Quantitative analysis of NOS2 positive macrophages in border areas of hearts in **D**. Student’s *t* test (n = 5).

**
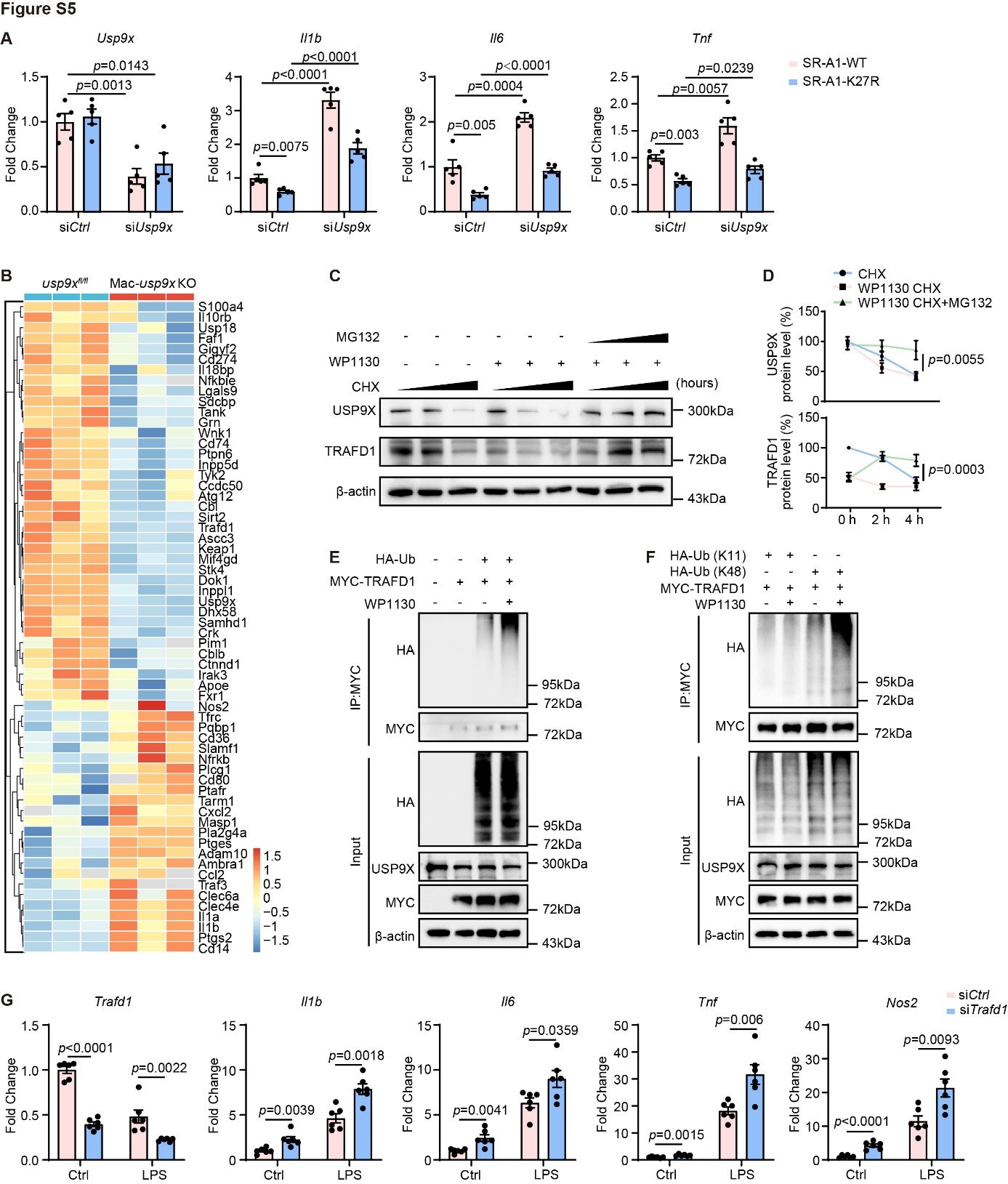
Figure S5: USP9X regulates macrophage-mediated inflammatory responses through stabilizing TRAFD1**

(**A**) Quantitative PCR analysis of indicated gene expressions in RAW264.7 cells stably overexpressing SR-A1-WT-EGFP or SR-A1-K27R-EGFP. Cells were transfected with either si*Ctrl* or si*Usp9x* for 48 h and subsequently exposed to either LPS (100 ng/mL) or PBS for another 24 h. Target gene expression was normalized to *Actb* mRNA levels. Two-way ANOVA with Tukey’s multiple comparisons test (n = 5). (**B**) Heatmap of differentially expressed proteins involved in inflammatory signaling pathways, identified in the proteomic analysis of USP9X-deficient versus control BMDMs. (**C**) BMDMs were first pretreated with or without WP1130 for 12h, followed by LPS treatment for 12 h. Subsequently, cells were incubated with CHX (50 μg/mL) or MG132 (10 μM) for the indicated times (0, 2, 4 h). WB analysis was performed to assess endogenous TRAFD1 and USP9X levels. (**D**) Quantification of TRAFD1 protein turnover rate from **C**. Protein levels were normalized to levels at time zero (0 h) in the control group. Two-way ANOVA with Tukey's multiple comparisons test (n = 3). (**E**) HEK293 cells were co-transfected with HA-Ub and MYC-TRAFD1 for 48 h, followed by treatment with or without WP1130 for 24 h and treated with MG132 for the final 2 h. Cells were subjected to IP using MYC magnetic beads, followed by immunoblotted with indicated antibodies (n = 3). (**F**) HEK293 cells were co-transfected with MYC-TRAFD1, along with either HA-Ub (K11), or HA-Ub (K48) for 48 h, then treated with or without WP1130 for 24 h and MG132 for the last 2 h. Cells were subjected to IP using MYC magnetic beads, followed by immunoblotted with indicated antibodies (n = 3). (**G**) Quantitative PCR analysis of indicated gene expressions in BMDMs. Cells were transfected with either si*Ctrl* or si*Trafd1* for 48 h and subsequently exposed to either LPS (100 ng/mL) or PBS for another 24 h. Gene expression levels were normalized to *Actb* mRNA. Student’s *t* test (n = 5).

**
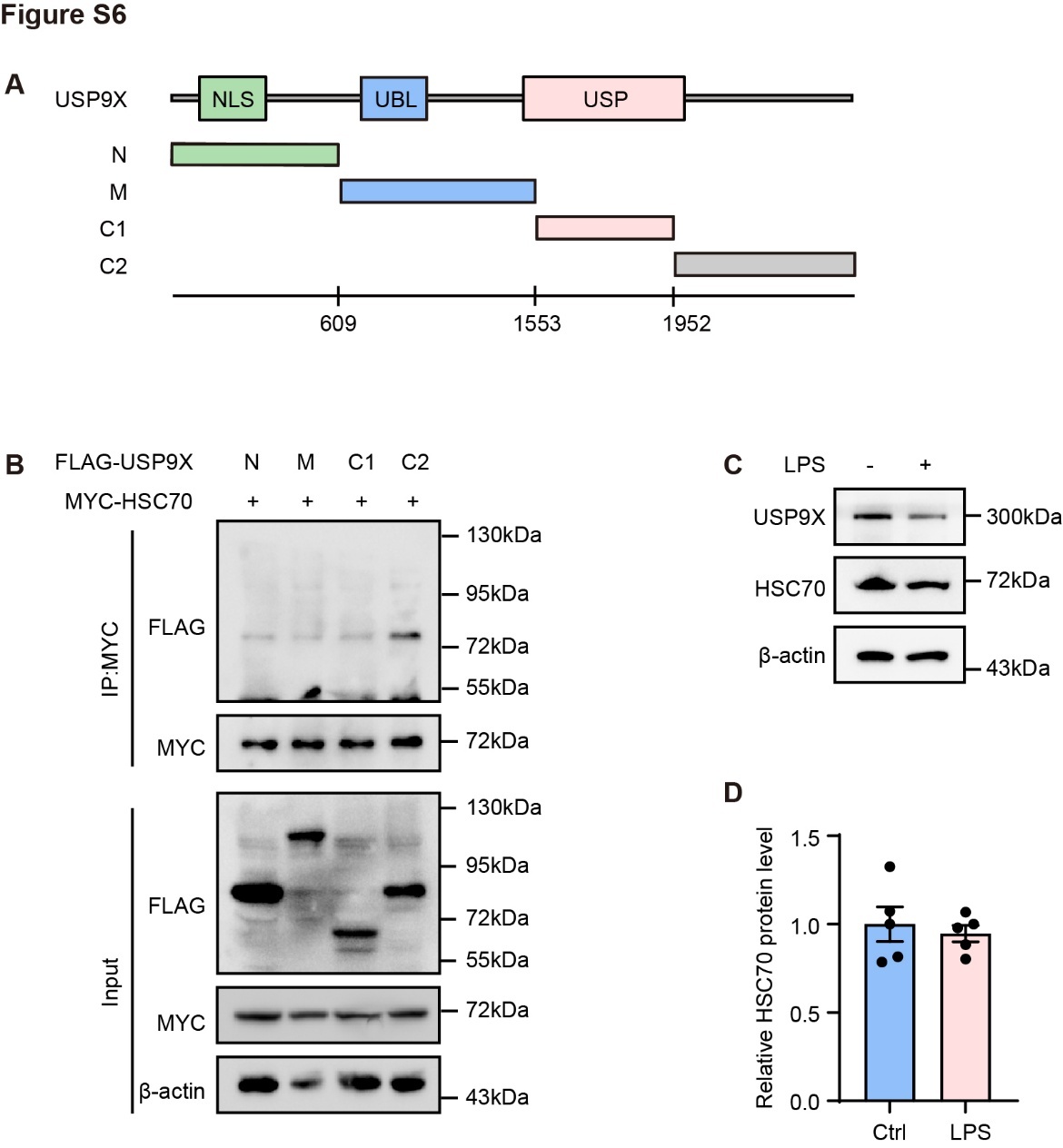
**

**Figure S6**

 (**A**) Schematic diagram of USP9X domain structure, showing the functional nuclear localization signal, ubiquitin-like and ubiquitin-specific protease domains and the four generated truncation mutants (N, M, C1, C2). (**B**) HEK293 cells were co-transfected with constructs expressing the FLAG-tagged USP9X truncation variants (N, M, C1, C2) and MYC-HSC70. WCL were subjected to IP using anti-MYC magnetic beads, followed by WB analysis of the indicated proteins (n = 3). (**C**) BMDMs were treated with vehicle or LPS. Cell lysates were analyzed by WB. (**D**) Quantification of USP9X protein expression shown in **C**. Student’s *t* test (n = 5).

**
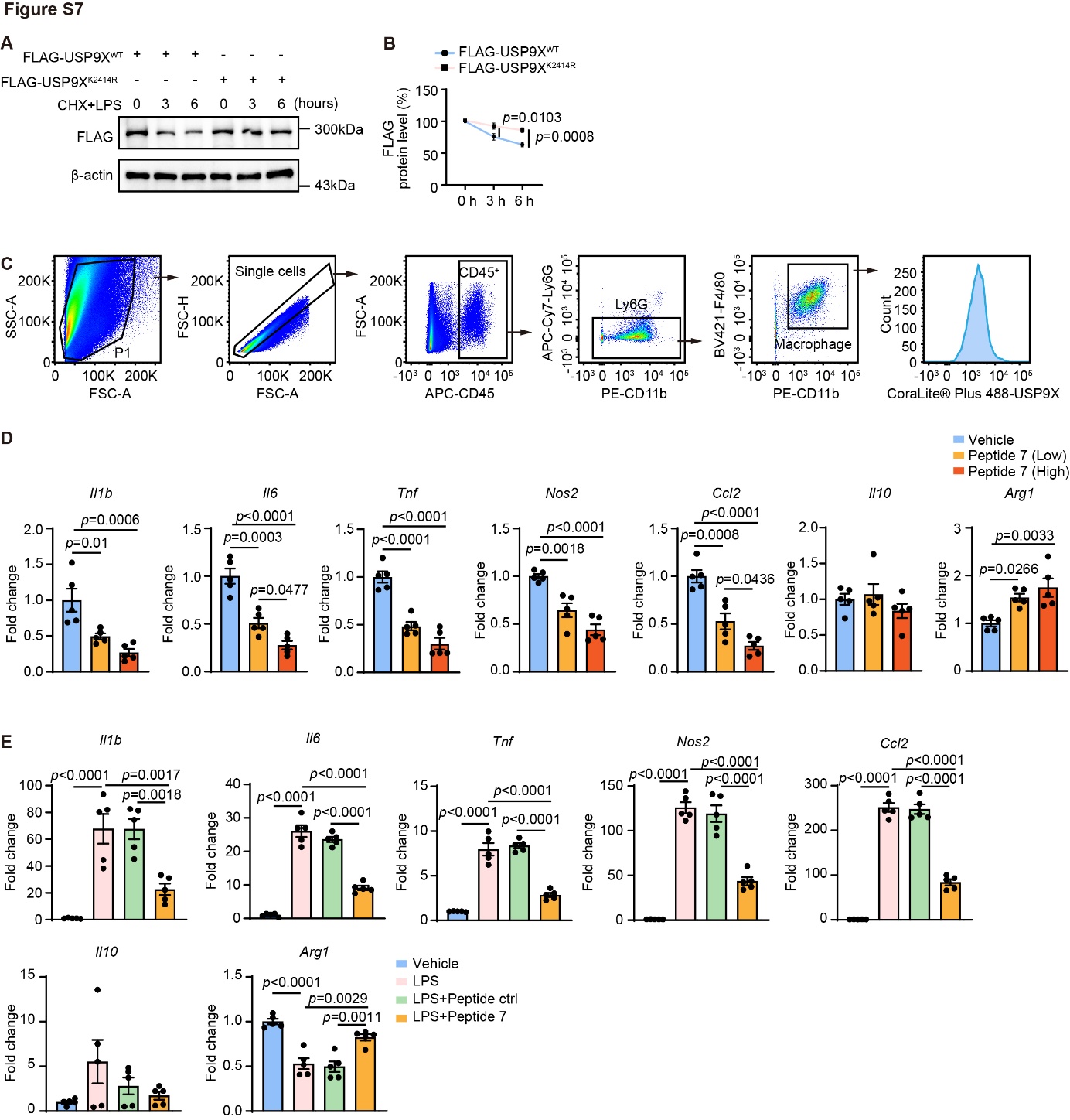
**

**Figure S7:**

(**A**) BMDMs were transduced with lentiviral vectors encoding FLAG-USP9X^WT^ or FLAG-USP9X^K2414R^ for 48 h, followed by treatment with CHX (25 μg/mL) and LPS for the indicated time (0, 3, 6 h). WB analysis was performed to assess the levels of FLAG. (**B**) Quantification of FLAG-USP9X protein stability from **A**. Protein levels at each time point were normalized to the level at 0 h in the control group. Student’s *t* test (n = 4). (**C**) Flow cytometry gating strategy for identifying cardiac macrophages from mice at 0- or 3-days post-MI. (**D**) Quantitative PCR analysis of indicated gene expression in heart tissues. Mice were treated with a low dose (20 mg/kg/day) or a high dose (40 mg/kg/day) of peptide 7 via intraperitoneal injection (100 µL per dose, administered starting at day 0 and continuing every two days). Gene expression levels were normalized to *Actb* mRNA. One-way ANOVA with Tukey’s multiple comparisons test (n = 5). (**E**) Quantitative PCR analysis of indicated gene expression in BMDMs isolated from WT mice treated as indicated for 24 h. Target gene expression was normalized to *Actb* mRNA levels. One-way ANOVA with Tukey multiple comparisons test (n = 5).


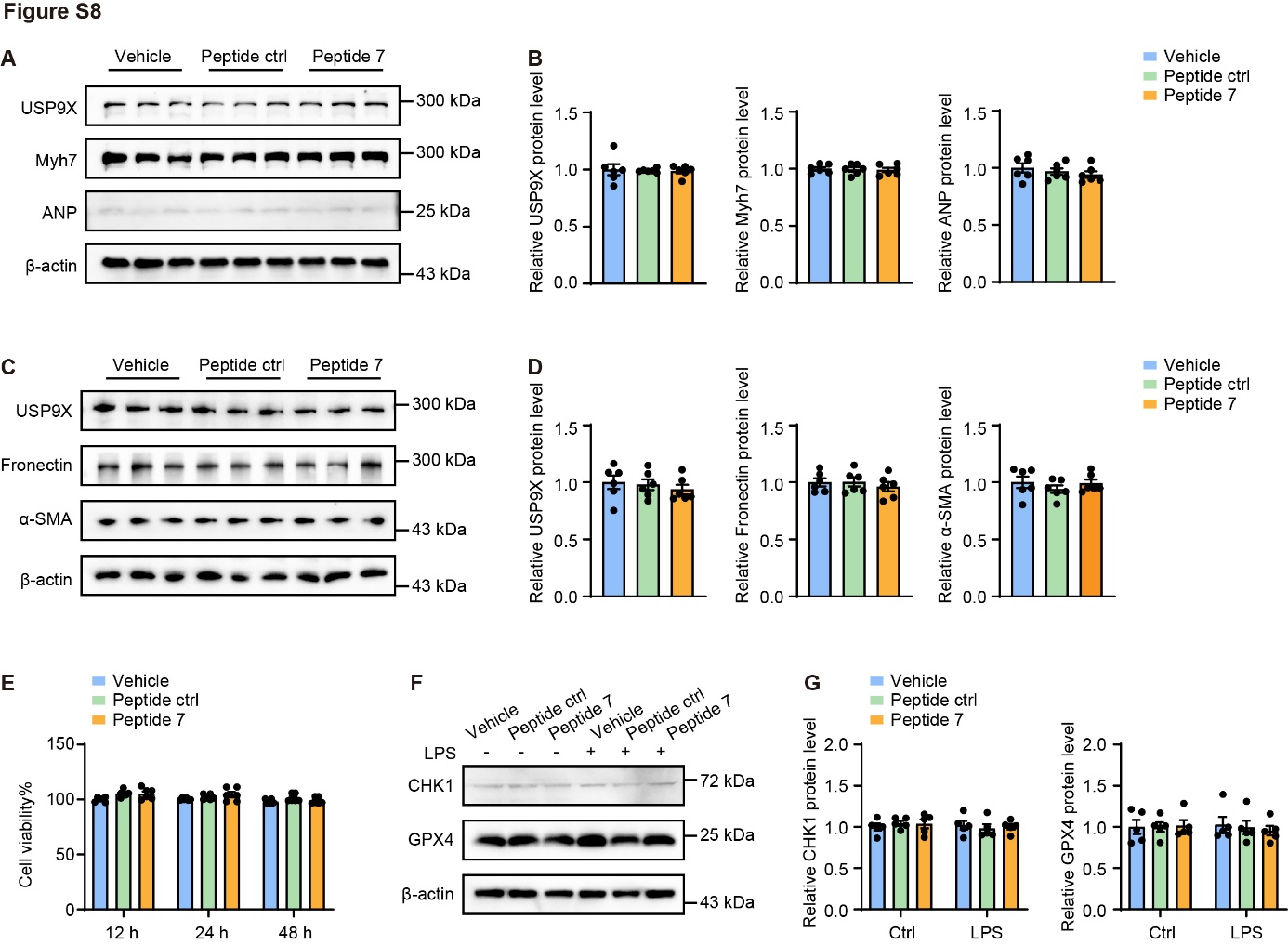


**Figure S8:**

(**A**) Neonatal mouse cardiomyocytes were treated with indicated peptides, followed by WB analysis of the indicated proteins. (**B**) Quantification of indicated proteins expression in **A**, One-way ANOVA with Tukey multiple comparisons test (n = 6). (**C**) Cardiac fibroblasts were treated with indicated peptides, followed by WB analysis of the indicated proteins. (**D**) Quantification of indicated proteins expression in **C**. One-way ANOVA with Tukey multiple comparisons test (n = 6). (**E**) Cardiomyocytes were treated with 20 μM indicated peptides for 12, 24, and 48 h, followed by MTT assay. No significant reduction in viability was observed at any time point. Two-way ANOVA with Tukey multiple comparisons test (n = 6). (**F**) BMDMs were incubated with the indicated peptides in the presence or absence of LPS for 24 h. WCL were immunoblotted for the indicated CMA substrates. (**G**) Quantification of proteins expression in **F**, One-way ANOVA with Tukey multiple comparisons test (n = 5).


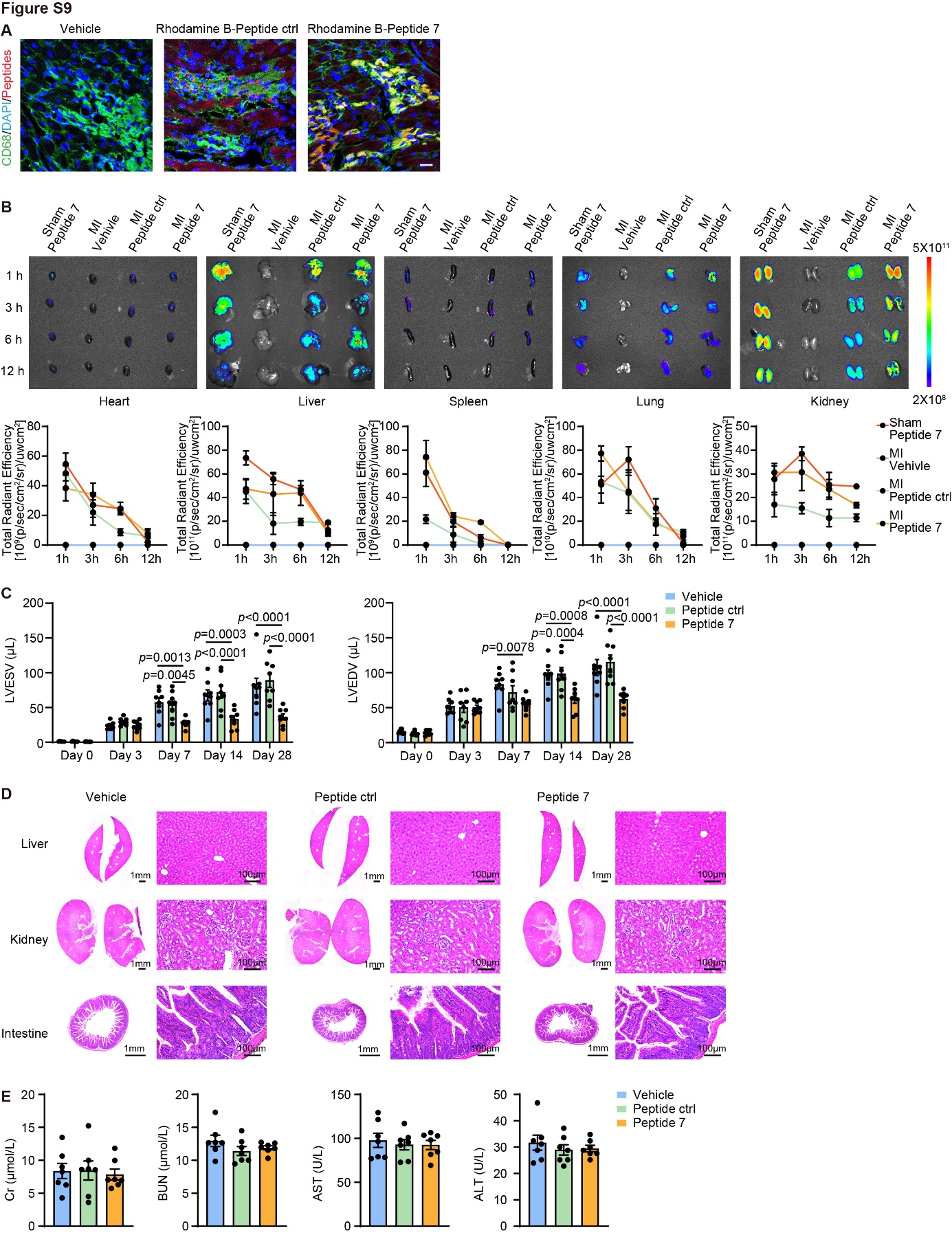


**Figure S9:**

(**A**) Immunofluorescence staining for CD68 (green) and DAPI (blue) in heart cryosections. Sections were obtained from mice at 3 days MI following treatment with vehicle or the indicated peptides. Scale bar, 20 μm. (**B**) Sham-operated or MI mice were injected with Cy5.5-conjugated peptides at 1 day post-surgery. Major organs were harvested at 1-, 3-, 6-, and 12-hours post-injection for ex vivo fluorescence imaging. (Upper) Representative fluorescence images of organs from different treatment groups. (Lower) Quantitative analysis of fluorescence intensity normalized to background. (**C**) Echocardiographic measurements of LVESV and LVEDV in indicated groups on day 0, 3, 7, 14 and 28 post-MI. Two-way ANOVA with Tukey's multiple comparisons test (n = 7). (**D**) Histopathological analysis of H & E-stained liver, kidney and intestine from mice treated with vehicle or different peptides 28 days post-MI. Scale bars as indicated. (**E**) Indices of hepatic and renal function in indicated groups on day 28 after MI. Cr, BUN, ALT and AST. One-way ANOVA with Tukey's multiple comparisons test (n = 7).

**Table S1. List of peptide sequences used in this study**

| Name | Sequences |
| --- | --- |
| Peptide 1 | YGRKKRRQRRRQLEKI |
| Peptide 2 | YGRKKRRQRRRVIKEQ |
| Peptide 3 | YGRKKRRQRRRERFFQ |
| Peptide 4 | YGRKKRRQRRRFDRLQ |
| Peptide 5 | YGRKKRRQRRRQRIRD |
| Peptide 6 | YGRKKRRQRRRQKIIE |
| Peptide 7 | YGRKKRRQRRRDLKRQ |

**Table S2. List of gene-specific primer sequences used in this study**

| Target | | Primer |
| --- | --- | --- |
| *Actb* | Forward | GGCACCACACCTTCTACAATG |
|  | Reverse | GGGGTGTTGAAGGTCTCAAAC |
| *Usp9x* | Forward | GTAAACTGGGGCTTTGCCCA |
|  | Reverse | TCAATGAAAAGAAACAAAGCCCAT |
| *Tnf* | Forward | ATGAGAAGTTCCCAAATGGC |
|  | Reverse | CTCCACTTGGTGGTTTGCTA |
| *Il1b* | Forward | GAAGAAGAGCCCATCCTCTG |
|  | Reverse | TCATCTCGGAGCCTGTAGTG |
| *Il6* | Forward | AGTCCGGAGAGGAGACTTCA |
|  | Reverse | TTCCACGATTTCCCAGAG |
| *Ccl2* | Forward | GAAGGAATGGGTCCAGACAT |
|  | Reverse | ACGGGTCAACTTCACATTCA |
| *Nos2* | Forward | CCCTTCCGAAGTTTCTGGCAGC |
|  | Reverse | GGCTGTCAGAGCCTCGTGGCTTTGG |
| *Il10* | Forward | CCCAGAAATCAAGGAGCATT |
|  | Reverse | TCACTCTTCACCTGCTCCAC |
| *Arg1* | Forward | CAGTGGCTTTAACCTTGGCT |
|  | Reverse | GTCAGTCCCTGGCTTATGGT |

**Table S3. Antibodies used for flow cytometric cell sorting**

| Target | Conjugate | Supplier | Catalog Number |
| --- | --- | --- | --- |
| CD45 | APC | Biolegend | 103112 |
| CD45 | PercP-Cy5.5 | Biolegend | 103132 |
| CD11b | PE | Biolegend | 101207 |
| Ly6G | APC-Cy7 | Biolegend | 560600 |
| F4/80 | PerCP-Cy5.5 | BD | 567202 |
| F4/80 | BV421 | BD | 565411 |
| USP9X | CoraLite® Plus 488 | Proteintech | CL488-55054 |
| CCR2 | APC-Cy7 | Biolegend | 150641 |
| MHC-II | APC | Sino Biological | 11091-MM01-A |

**References**

[1] C. Zhang, T. Zhou, Z. Chen, M. Yan, B. Li, H. Lv, C. Wang, S. Xiang, L. Shi, Y. Zhu, D. Ai, Coupling of Integrin α5 to Annexin A2 by Flow Drives Endothelial Activation, Circulation research. 127 (2020) 1074-1090. <https://doi.org/10.1161/circresaha.120.316857>.

[2] F. Yao, P. Yu, Y. Li, X. Yuan, Z. Li, T. Zhang, F. Liu, Y. Wang, Y. Wang, D. Li, B. Ma, C. Shu, W. Kong, B. Zhou, L. Wang, Histone Variant H2A.Z Is Required for the Maintenance of Smooth Muscle Cell Identity as Revealed by Single-Cell Transcriptomics, Circulation. 138 (2018) 2274-2288. <https://doi.org/10.1161/circulationaha.117.033114>.
